# Complex molecular mechanisms underlying seedling salt tolerance in rice revealed by comparative transcriptome and metabolomic profiling

**DOI:** 10.1093/jxb/erv476

**Published:** 2015-10-27

**Authors:** Wen-Sheng Wang, Xiu-Qin Zhao, Min Li, Li-Yu Huang, Jian-Long Xu, Fan Zhang, Yan-Ru Cui, Bin-Ying Fu, Zhi-Kang Li

**Affiliations:** ^1^Institute of Crop Sciences/National Key Facility for Crop Gene Resources and Genetic Improvement, Chinese Academy of Agricultural Sciences, Beijing 100081, PR China; ^2^School of Science, Anhui Agricultural University, Hefei 230036, PR China; ^3^Shenzhen Institute of Breeding and Innovation, Chinese Academy of Agricultural Sciences, Shenzhen 518120, PR China

**Keywords:** ABA, metabolomics, molecular mechanisms, *Oryza sativa* L, salt tolerance, transcriptome.

## Abstract

To understand the physiological and molecular mechanisms underlying seedling salt tolerance in rice (*Oryza sativa* L.), the phenotypic, metabolic, and transcriptome responses of two related rice genotypes, IR64 and PL177, with contrasting salt tolerance were characterized under salt stress and salt+abscisic acid (ABA) conditions. PL177 showed significantly less salt damage, lower Na^+^/K^+^ ratios in shoots, and Na^+^ translocation from roots to shoots, attributed largely to better salt exclusion from its roots and salt compartmentation of its shoots. Exogenous ABA was able to enhance the salt tolerance of IR64 by selectively decreasing accumulation of Na^+^ in its roots and increasing K^+^ in its shoots. Salt stress induced general and organ-specific increases of many primary metabolites in both rice genotypes, with strong accumulation of several sugars plus proline in shoots and allantoin in roots. This was due primarily to ABA-mediated repression of genes for degradation of these metabolites under salt. In PL177, salt specifically up-regulated genes involved in several pathways underlying salt tolerance, including ABA-mediated cellular lipid and fatty acid metabolic processes and cytoplasmic transport, sequestration by vacuoles, detoxification and cell-wall remodeling in shoots, and oxidation–reduction reactions in roots. Combined genetic and transcriptomic evidence shortlisted relatively few candidate genes for improved salt tolerance in PL177.

## Highlight

Comprehensive analyses of phenotypic, metabolic, and transcriptome data from two genotypes with contrasting salt tolerance provided a more complete picture of the molecular mechanisms underlying seedling tolerance in rice.

## Introduction

Rice (*Oryza sativa* L.) is the staple food crop for most Asian people. Grown in diverse ecologies of the world, rice plants suffer many abiotic stresses, of which salt is the most important constraint limiting rice yield in the coastal regions of the tropics and the inland arid/semi-arid areas (Ondrasek *et al.*, 2011). Developing salt-tolerant rice varieties is the most efficient way to reduce the yield loss caused by salt.

Rice is sensitive to salt stress at both the young seedling and reproductive stages (Lutts *et al.*, 1995), and rice genotypes vary considerably in their tolerance to salt, which involves complex physiological mechanisms (Li and Xu, 2007) and is controlled by many quantitative trait loci (QTLs) (Singh *et al.*, 2007; Zang *et al.*, 2008; Thomson *et al.*, 2010; Wang *et al.*, 2013). Several major salt tolerance QTLs with relatively large effects such as *qSKC1*, *qSNC7*, and *Saltol* have been identified (Lin *et al.*, 2004; Thomson *et al.*, 2010). *qSKC1* is a sodium transporter controlling K^+^ homeostasis in rice under salt stress (Ren *et al.*, 2005). Unfortunately, few successful cases have been reported in developing new salt-tolerant rice varieties by using molecular breeding approaches based on these identified salt tolerance QTLs.

Historically, plant salt tolerance was found to be associated with the accumulation of specific metabolites in plant tissues such as proline and trehalose (Ashrafa and Foolad, 2007; Li *et al.*, 2011). At the transcriptomic level, plant responses to salt are known to involve large numbers of genes functioning in stress signaling, transcription regulation, ion transport, and biosynthesis of specific metabolites of complex signaling pathways (Kawasaki *et al.*, 2001; Walia *et al.*, 2007; Cotsaftis *et al.*, 2011; Kumar *et al.*, 2013). As an important phytohormone in plant growth and development, abscisic acid (ABA) is known to be involved in plant abiotic stress tolerances (Ye *et al.*, 2012). Exogenous ABA application was able to offset the osmotic and ion stress effects from salt stress in common bean (Khadri *et al.*, 2007), wheat (Gurmani *et al.*, 2013), and rice (Sripinyowanich *et al.*, 2013) by reducing the sodium concentration and improving osmotic adjustment. According to physiological evidence, rice shoots were more stressed than roots under salt (Cotsaftis *et al.*, 2011). At the transcriptomic level, there was limited overlap of stress-responsive genes between shoots and roots of Arabidopsis (Kreps *et al.*, 2002). However, there is little information regarding the relationship between the metabolomic responses and the transcriptomic responses to salt stress in rice, nor about the relationship between shoots and roots of rice, because most reported studies have focused on either shoots or roots of plants.

In this study, the transcriptome and metabolome profiles in the shoots and roots of two related rice genotypes, IR64 and PL177, with contrasting salt tolerance were investigated under salt stress and salt+ABA conditions. Our results revealed organ-specific transcriptome reprogramming and primary metabolite changes in the two genotypes in their response to the treatments, which provides insights into the molecular networks underlying salt tolerance in rice.

## Materials and methods

### Plant materials and stress treatment

A salt-tolerant F_6_ pyramiding line, PL177, derived from a cross between two IR64 introgression lines (Supplementary Figs S1 and S2 at *JXB* online) and its background parent, IR64, were used in this study. IR64 is moderately sensitive to salt stress (Kumari *et al.*, 2009). The seedlings were cultured in Yoshida solution in a box containing 40 plants of each genotype plus 20 plants of the salt-sensitive standard, IR29 (Yoshida *et al.*, 1976), for 11 d after germination. The salinity treatment was applied by adding NaCl in three steps to reach a final concentration of 140mM, as described by Walia *et al.* (2007) with minor modifications (Supplementary Fig. S3 at *JXB* online). The seedlings were independently subjected to one of three different treatments: no-stress control, salt stress (140mM NaCl), and salt+ABA (140mM NaCl+10 μM ABA), with three replications per treatment. The seedlings were grown in a phytotron with 29/22 °C day/night temperatures and a minimum relative humidity of 70%. The pH of the nutrient solution was adjusted daily to 5.5 by adding sulfuric acid and was refreshed every week.

### Phenotyping and physiological characterization

The yield performances of the two genotypes under normal and salt stress conditions were evaluated in the summer of 2010. The two genotypes were grown in 2 m^2^ plots with three replications for each genotype under normal field conditions in an experimental farm of the Institute of Crop Sciences, Chinese Academy of Agricultural Sciences, Beijing. Under the salt stress conditions, the two genotypes were grown in three concrete ponds at the Tianjing Academy of Agricultural Sciences, which were irrigated weekly with salty water (0.8%) from the seedling stage (7 d after transplanting) until maturity. At maturity, grain yield and related traits were measured from 12 plants of each genotype.

After 11 d under three different treatments, plants of PL177 and IR64 were visually scored for their symptoms of salt injury using the modified standard evaluation system (SES) for rice (Gregorio *et al.* 1997, Supplementary Table S1 at *JXB* online). Final scoring and sampling were done when the sensitive standard IR29 scored 7. Each sampled plant was then measured for its shoot and root lengths (cm). The dry weights of the root and shoot samples were measured after being oven dried at 70–75 °C for 72h. Relative water content (RWC) of each plant was determined as a percentage using the equation: RWC (%)=(FW – DW)/(TW– DW)×100, where FW, DW, and TW are fresh weight, dry weight and total weight of each plant (Larbi and Mekliche, 2004). The sodium and potassium concentrations and sodium/potassium ratio (Na^+^/K^+^) in roots and shoots were measured according to the method described by Zang *et al.* (2008).

### Metabolite profiling and data analysis

Metabolites from the sampled shoots and roots of both genotypes of each replication under the different treatments were extracted and measured based on their chromatograms and mass spectra using the GC-MS Postrun Analysis (Shimadzu) software as described previously (Zhao *et al.* 2013). Specific mass spectral fragments were detected in defined retention-time windows using the mass spectral libraries of NIST08, NIST08S, and Wiley 9 in the public domain mass spectra library of the Max Planck Institute in Germany (http://csbdb.mpimp-golm.mpg.de/csbdb/). The quantification of each metabolite was based on its specific peak area. Further confirmation of most identified amino acids, organic acids, and sugars was performed by standard addition experiments using the pure authenticated compounds. In total, 88 primary metabolites were identified in the present study (Supplementary Table S2 at *JXB* online).

### Total RNA isolation and microarray analysis

Total RNA was extracted from liquid-nitrogen-frozen shoot and root samples using TRIzol reagent, and purified and concentrated using an RNeasy MinElute Cleanup kit (Qiagen). Two micrograms of total RNA was used for microarray analysis by CapitalBio Corporation (Beijing) as described previously (Wang *et al.*, 2011). Differentially expressed genes (DEGs) were identified using the empirical criterion of a greater than 2-fold change and a significant *q* value (false discovery rate-adjusted *P* value) of <0.05 based on three independent biological replicates. Cluster analyses of the identified DEGs were performed using the complete linkage hierarchical analysis of TIGR MeV 4.2 software (http://www.tm4.org). Gene Ontology (GO) enrichment/over-representation analysis was performed using AgriGO (http://bioinfo.cau.edu.cn/agriGO/; Du *et al.*, 2010). Metabolic pathways analysis was carried out using MapMan software (version 3.5.1R2) (http://mapman.gabipd.org/; Thimm *et al.*, 2004)

### Real-time PCR confirmation of salt-responsive genes

A set of 10 DEGs was selected to confirm the expression level of microarray results using real-time-PCR (RT-PCR). The sequences corresponding to the genes were obtained from the rice genome sequences database (TIGR), and the sequences of exons of each gene were used to design the RT-PCR primers using Primer3 software (http://frodo.wi.mit.edu/); the *Actin* gene was used as the internal control. The same RNA samples for the microarray analysis were used for RT-PCR, as described previously (Wang *et al.* 2011).

### Data availability

The entire set of original microarray data has been deposited in NCBI’s Gene Expression Omnibus under GEO Series number GSE58603.

## Results

### Phenotypic and genotypic differences in salt tolerance, yield, and related traits under salt and salt+ABA treatments

**Fig. 1.**
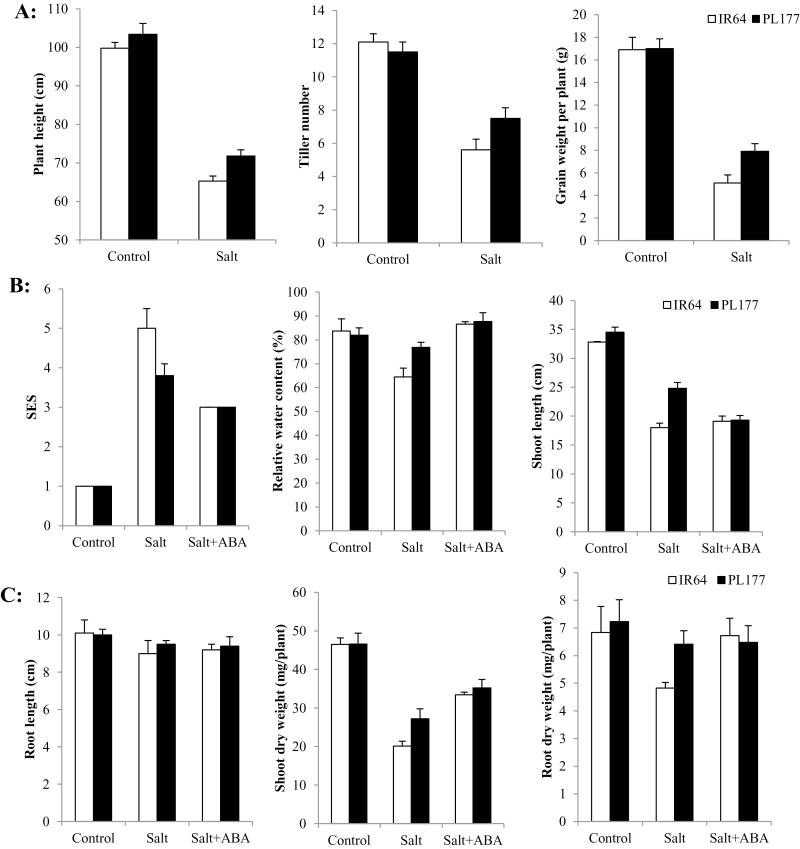
Morphological and yield trait performance of IR64 and PL177 measured under control, salt, and/or salt+ABA conditions. Results are shown as means±SD.

Under normal field conditions, PL177 showed no difference from IR64 for yield and related traits except for having a slightly increased height by 3.6cm (Fig. 1A, Supplementary Table S3 at *JXB* online). Under consistent salt stress from the seedling stage to maturity, PL177 was 6.5cm taller with 1.9 (33.9%) more tillers and 2.8g (54.9%) higher yield per plant than IR64, indicating that the improved yield of PL177 under salt stress was due primarily to its improved seedling salt tolerance.

Significant differences were detected between IR64 and PL177 for salt damage scores and related traits measured under all three treatments at the seedling stage (Fig. 1B, 1C, Supplementary Table S3). Under the non-stress control and salt+ABA treatments, no significant differences were observed between IR64 and PL177 for all measured traits. However, under salt stress, salt tolerance-related trait values of both genotypes were significantly reduced but to a significantly lesser degree for PL177 than for IR64. Exogenous ABA significantly improved the trait values of IR64 for shoot dry weight (SDW), root dry weight (RDW) and RWC, but not for plant height (PH) and root length (RL). For PL177, exogenous ABA also improved its salt tolerance, SDW, and RWC, but caused no difference in RL and reduced its PH. These results indicated that exogenous ABA could remarkably offset the detrimental effect of salt stress on seedling growth of IR64.

**Fig. 2.**
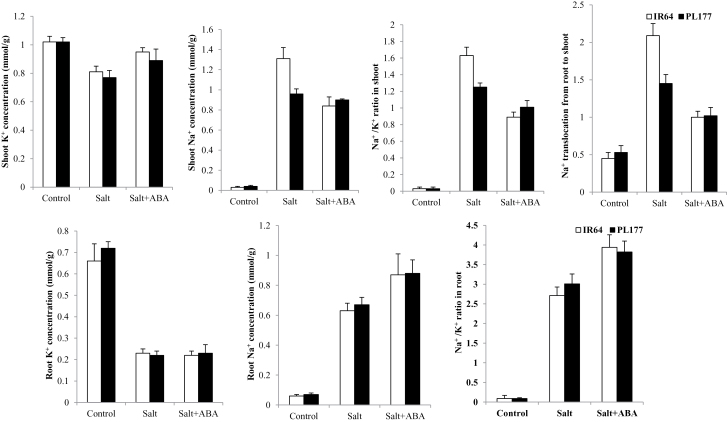
Shoot and root Na^+^ and K^+^ concentrations and Na^+^/K^+^ ratio in IR64 and PL177 under control, salt, and salt+ABA treatment conditions. Results are shown as means±SD.

In the absence of NaCl and ABA, IR64 and PL177 showed no differences in Na^+^ and K^+^ concentrations in shoots and roots (Fig. 2, Supplementary Table S4 at *JXB* online). Salt stress significantly increased Na^+^ concentrations and Na^+^/K^+^ ratios in shoots and roots of both genotypes. However, the genotypic differences in Na^+^ and K^+^ concentrations under salt were more evident in shoots than in roots, indicating a significant difference between IR64 and PL177 in shoot Na^+^ translocation. Compared with IR64, PL177 showed significantly lower Na^+^/K^+^ ratios in shoots, attributed largely to more Na^+^ translocation from the roots to the shoots in IR64. Thus, the improved salt tolerance of PL177 was at least partially attributed to its much lower Na^+^ concentration in shoots and thus lower Na^+^ toxicity in its shoots compared with IR64.

The salt+ABA treatment had an overall mitigation effect on salt stress. In comparison with the salt-stressed rice seedlings, the shoot K^+^ content in both genotypes was significantly increased by exogenous ABA. ABA also increased the root Na^+^ concentration but had no effect on the root K^+^ concentration in both genotypes (Fig. 2, Supplementary Table S4). However, under salt stress, exogenous ABA significantly reduced the shoot Na^+^ concentrations but increased root Na^+^ concentrations of both genotypes, although this reduction or increase was more evident in IR64 than in PL177. As a result, the Na^+^/K^+^ ratio was remarkably reduced in shoots and increased in roots in both genotypes. The translocation of Na^+^ from the roots to the shoots was significantly reduced in both genotypes by exogenous ABA, but the genotypic difference in Na^+^/K^+^ ratio remained significant although much smaller compared with the salt treatment without ABA.

### Genotypic differences in metabolite profiling under salt and salt+ABA

**Fig. 3.**
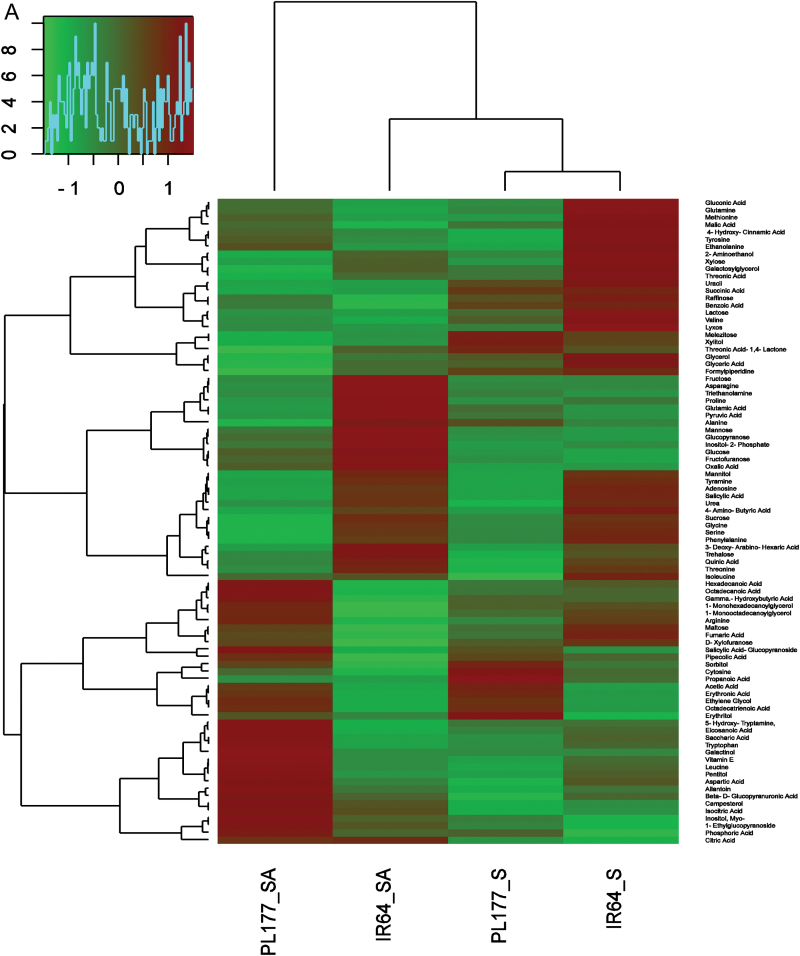

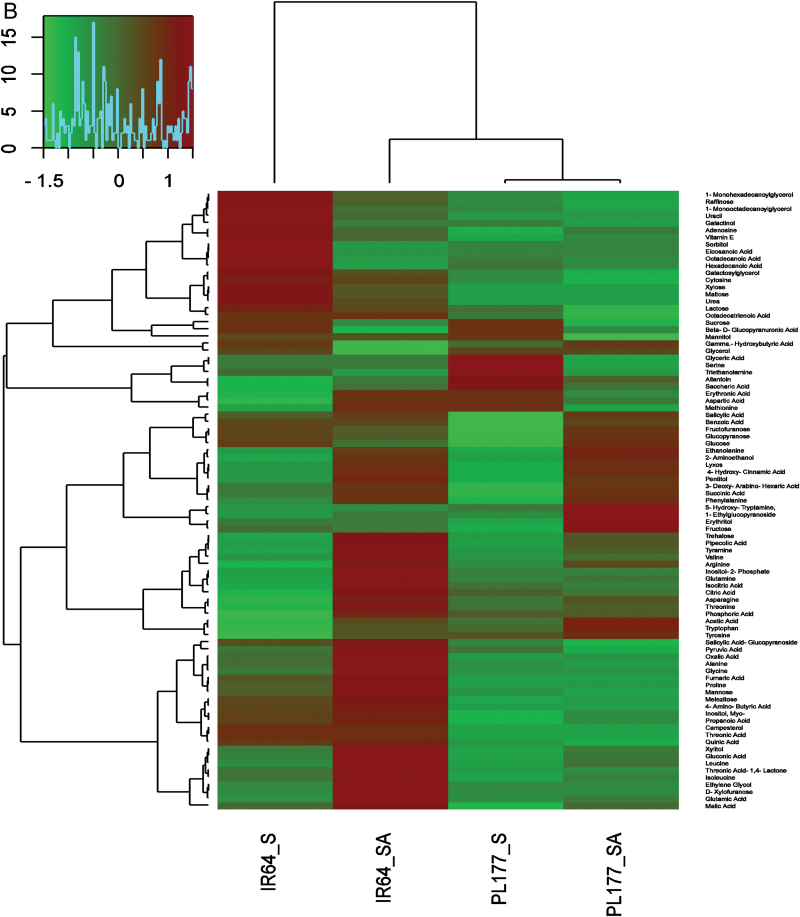
Results of hierarchical cluster analysis of changed metabolite pools. Hierarchical trees were drawn based on detected changed metabolites in shoots (A) and roots (B) of IR64 and PL177 under salt stress (S), salt stress+ABA (SA) treatments. (This figure is available in color at *JXB* online.)

Figure 3 and Supplementary Tables S5 and S6 at *JXB* online show the general pattern of the relative changes of the 88 measured primary metabolites (19 amino acids, 24 sugars, 29 organic acids, and 16 other small molecular components) in shoots and roots of PL177 and IR64 in response to the salt stress and salt+ABA treatments. In general, the level of virtually all measured primary metabolites except for 5-hydroxytryptamine, trehalose, and allantoin, was significantly higher in shoots than in roots of both genotypes and these changes were highly organ specific.

In shoots, 38 of the 88 measured metabolites showed significant genotypic changes (G) in response to the treatments and explained an average of 6.5% of the total phenotypic variation; the treatments (T) caused significant changes (mostly increases) in most (77/88) metabolites and explained an average 58.6% of the total phenotypic variation; the G×T interaction was significant for 32 of the measured metabolites and accounted for an average of 12.7% of the total variation (Supplementary Table S6A). According to the principal component (PC) analyses, PC1 explained 60.1% of the total measured metabolite variation in the direction of the treatments, while PC2 accounted for 19.4% of the total variation that was attributed partially to the treatment differences and partially to the genotypic differences under salt+ABA. PC3 explained 11.3% of the total variation, which was due primarily to the genotypic differences within different treatments (Supplementary Table S7 at *JXB* online and Fig. 3A).

Supplementary Table S8 at *JXB* online shows those metabolites that increased significantly under salt and/or salt+ABA treatment in one or both genotypes; these could be classified into two major types based on their differential responses to the salt and salt+ABA treatments. Type I included 20 metabolites in shoots that increased significantly by more than 2.5-fold in one or both genotypes in response to salt but were insensitive to ABA. Of these, adenosine increased most dramatically by >18-fold and 44-fold in PL177 and IR64, respectively. Type II included 16 metabolites that were significantly responsive to both salt and ABA. Of these, proline, glutamine, and fructose were the most notable. The proline content increased by 66-fold and 25-fold in PL177, and by 204-fold and 769-fold in IR64 under salt and salt+ABA, respectively. The glutamine content increased by 4.7-fold and 6.3-fold in PL177, and by 13.3-fold and 2.6-fold in IR64 under salt and salt+ABA, while the fructose content increased by 15-fold and 22-fold in PL177, and by 28-fold and 515-fold in IR64 under salt and salt+ABA, respectively. In particular, the accumulation in shoots of both genotypes under salt and/or salt+ABA was highest for proline, glutamine, fructose, and fructofuranose, indicating their unique roles in rice salt tolerance.

In roots, 32 of the 88 measured metabolites showed significant genotypic changes in response to the treatments and explained an average of 10.1% of the total phenotypic variation; the treatments caused significant changes in 58 of the 88 measured metabolites and explained an average 38.7% of the total phenotypic variation; the G×T interaction was significant for 37 of the measured metabolites and accounted for an average of 15.6% of the total variation (Supplementary Tables S5 and S6B). Again, the maximum direction (PC1) of the metabolite changes in roots was attributed primarily to the treatments, which explained 35.5% of the total measured metabolite variation, and partially to the genotypic difference within the salt+ABA treatment (Supplementary Table S7, Fig. 3B). PC2 accounted for 26.3% of the total variation and was attributed partially to the treatment differences and partially to the genotypic differences under salt stress. PC3 explained 16.9% of the total variation, which was due primarily to the genotypic differences within different treatments (Supplementary Table S7).

The number of metabolites and the overall levels of salt- or salt+ABA-induced metabolic changes were much less in roots than in shoots of both genotypes. In roots, allantoin showed extremely high accumulation in roots of both genotypes under salt and salt+ABA conditions, with its content increasing by 111-fold and 57.7-fold in PL177 under salt and salt+ABA, respectively, and by 41.8-fold in IR64 under salt+ABA, when compared with the non-stress control (Supplementary Tables S2 and S8). Urea was unique, which decreased slightly in PL177 but increased dramatically by 49 and 30 times in IR64 under salt and salt+ABA. The asparagine contents in PL177 and IR64 increased 7.8 and 2.3 times under salt and by 10.3 and 14.4 times under salt+ABA, respectively. The pentitol contents in PL177 and IR64 increased by 3.2 and 3.9 times under salt, and by 7.4 and 8.2 times under salt+ABA (Supplementary Table S8).

**Fig. 4.**
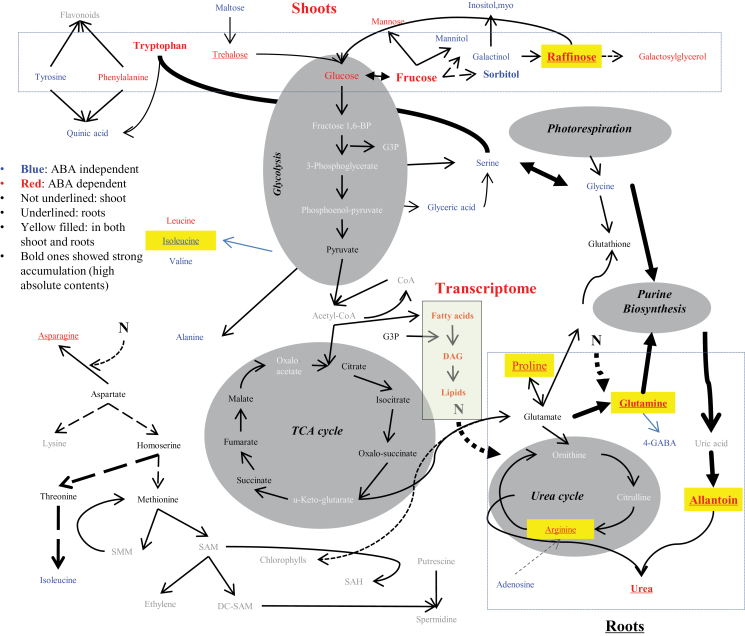
The metabolic pathways leading to the most primary metabolites under salt and salt+ABA conditions. (This figure is available in color at *JXB* online.)

Figure 4 shows the metabolic pathways leading to the most primary metabolites examined in this study, revealing two important results. First, salt-induced metabolic accumulation in the shoots of both genotypes primarily involved a few sugars (glucose, fructose, and fructofuranose) plus proline, all of which were ABA mediated. In contrast, salt-induced metabolic accumulation in the roots of both genotypes involved allantoin, urea, and glutamine, which are products of N metabolism. Trehalose was the only sugar with a relatively high accumulation in the roots of both genotypes.

### Global transcriptomic responses of PL177 and IR64 to salt and salt+ABA treatments

**Fig. 5.**
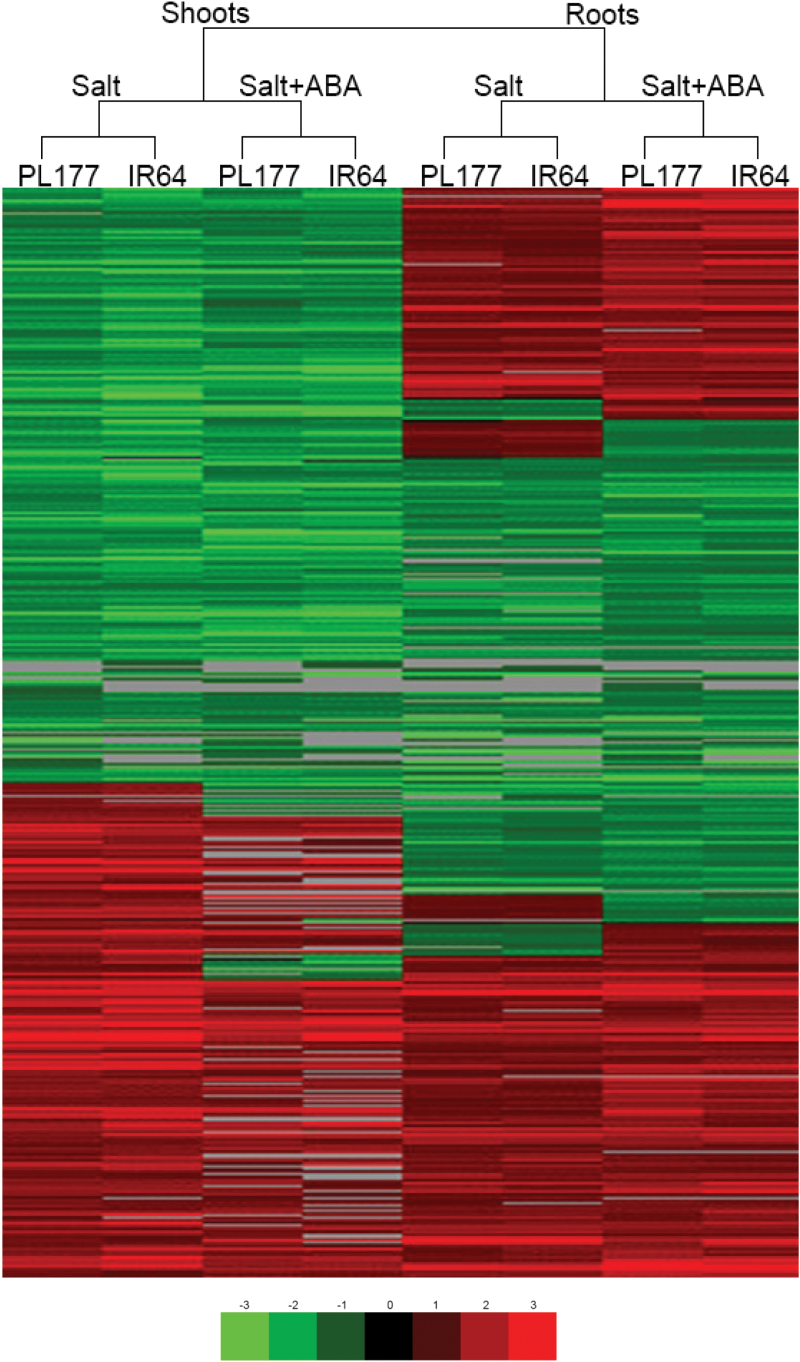
Cluster analysis of total DEGs under salt and salt+ABA treatments. (This figure is available in color at *JXB* online.)

Based on the criteria of a greater than 2-fold change and significance at *P*<0.05 in *t*-tests, a total of 3738 genes were found to be differentially regulated in at least one sample of the shoots and roots of PL177 and IR64 under salt and salt+ABA, when compared with their respective controls. Cluster analysis clearly differentiated these DEGs into two major clusters in shoots and roots, subclusters between the treatments, and subclusters between the two genotypes within each treatment (Fig. 5). Using 10 randomly selected genes and 160 comparisons of two organs in two genotypes under two treatments of salt versus the control (10×2×2×2×2), the quantitative RT-PCR results were in good agreement with those from the microarray experiments (*r* ^*2*^=0.741; Supplementary Fig. S4 and Table S9 at *JXB* online).

Table 1 summarizes the total number of up- and down-regulated genes in roots and shoots of both genotypes under two treatments, revealing two general patterns of transcriptomic responses of IR64 and PL177 to salt and salt+ABA. First, more genes were differentially regulated in roots by salt+ABA than by salt alone in both genotypes, but the opposite was true in shoots. Secondly, more genes were differentially regulated, particularly up-regulated, in the salt-tolerant line PL177 than in its salt-sensitive parent, IR64, under both the treatments.

**Table 1.** Summary of DEGs in seedlings of PL177 and IR64 under salt and salt+ABA conditions identified by the microarray experiments

Genotype	Organ	Treatment	Up-regulated	Down- regulated	Subtotal
IR64	Shoots	Salt	544	643	1187
		Salt+ABA	228	623	851
	Roots	Salt	525	362	887
		Salt+ABA	593	601	1193
PL177	Shoots	Salt	767	869	1636
		Salt+ABA	285	623	908
	Roots	Salt	653	307	960
		Salt+ABA	757	608	1365

### Genotype- and organ-specific gene expression changes in response to salt stress

**Fig. 6.**
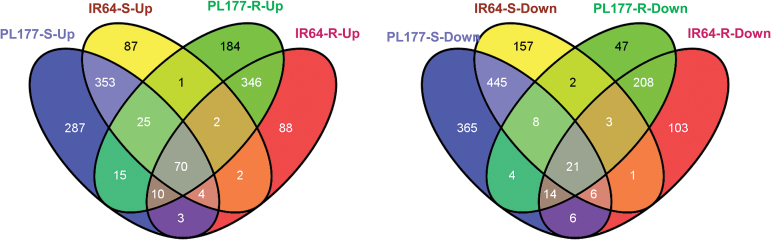
Four-way Venn diagram indicating the number of salt-up-regulated (left) and -down-regulated (right) genes found exclusively in the roots (R) and shoots (S) of two rice genotypes (salt-tolerant line PL177 and its salt-sensitive parent IR64) in the comparison between salt-stressed and non-stress treatments. (This figure is available in color at *JXB* online.)

Of the total 1636/1187 and 960/887 DEGs identified in the shoots and roots of PL177/IR64, respectively (Table 1), only 70 and 21 genes were commonly up- and down-regulated in the shoots and roots of both genotypes under salt stress (Fig. 6), indicating that the salt-induced transcriptomic responses of rice to salt stress were largely organ specific (Supplementary Table S10 at *JXB* online). The majority of DEGs in shoots or roots of both genotypes showed the same expression patterns in response to salt stress, which was expected from the fact that PL177 and IR64 share much of the same genetic background.

As shown in Fig. 5, many DEGs detected under salt were genotype specific, which should have contributed to the phenotypic differences in salt tolerance between PL177 and IR64. Compared with IR64, 287 and 365 genes were exclusively up- and down-regulated in shoots of PL177, while 184 and 47 genes were exclusively up- and down-regulated in the roots of PL177 under salt stress (Fig. 6). The 347 specifically up-regulated genes in the PL177 shoot were involved in eight pathways that are known to be associated with salt tolerance in plants (Supplementary Table S11 at *JXB* online). MapMan software was used to assign genes to functional categories (Supplementary Fig. S5 at *JXB* online). These pathways included cytoplasmic vesicle (GO:0031410), vesicle (GO:0031982), cytoplasmic membrane-bounded vesicle (GO:0016023), membrane-bounded vesicle (GO:0031988), hydrolase activity (GO:0016798), polysaccharide metabolism process (GO:0005976), hydrolase activity, hydrolyzing *O*-glycosyl compounds (GO:0004553), and the extracellular region (GO:0005576). These results suggested that sequestration by vacuoles, detoxification, and cell-wall remodeling are key mechanisms in shoots that contribute to the improved salt tolerance of PL177 (Supplementary Fig. S5A). There were only 36 genes that were specifically up-regulated in PL177 roots, which were involved in four major pathways, including the monocarboxylic acid metabolic process (GO:0032787), oxidoreductase activity (GO:0016491), and mono-oxygenase activity (GO:0004497), implying enhanced oxidation–reduction reactions in the roots of PL177 under salt conditions. Surprisingly, six genes involved in photosynthesis (GO:0015979) were also up-regulated in PL177 roots, suggesting their possible roles other than photosynthesis (Supplementary Fig. S5B).

Large numbers of genes of diverse functions were specifically down-regulated in the PL177 shoots (Supplementary Table S11). The most over-represented GO categories of these included catalytic activity (GO:0003824), kinase activity (GO: 0016301), transferase activity (GO: 0016740), photosynthetic membrane (GO: 0034357), and membrane (GO:0016020), showing that energy metabolism and photosynthesis were evidently repressed in the shoots of PL177.

By mapping the DEGs that were up-regulated exclusively in PL177 on the rice genome, we were able to identify 43 candidate genes that fell into the six genomic regions of PL177 introgressed from its donors (Supplementary Fig. S2 and Supplementary Table S12 at *JXB* online). Of these, several genes of regulatory function were of great interest, including an ABA-responsive element binding protein (Os06g0211200), a Zn-finger, a RING domain-containing protein (Os04g0105100), Annexin p33 (Os06g0221200), and a receptor kinase-like protein (LOC_Os11g07140).

### Identification of ABA-mediated pathways for salt tolerance in rice

**Fig. 7.**
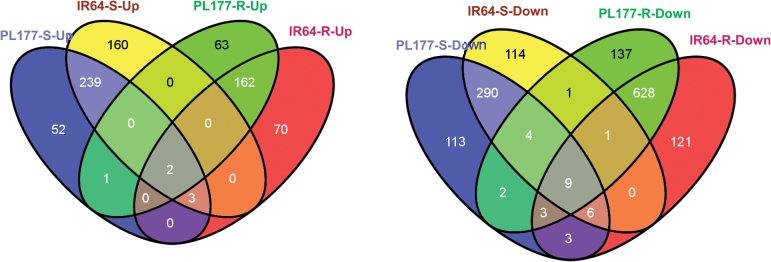
Four-way Venn diagram indicating the number of up-regulated (left) and down-regulated (right) genes found exclusively in the roots (R) and shoots (S) of two rice genotypes (salt-tolerant line PL177 and its salt-sensitive parent IR64) in the comparison between salt+ABA treatment and salt-stressed conditions. (This figure is available in color at *JXB* online.)

Because exogenous ABA was able to ameliorate the salt stress effect on both rice genotypes, direct comparison between the transcriptomic data under salt and salt+ABA conditions allowed us to determine DEGs and their involved pathways contributing to enhanced salt tolerance in rice. Figure 7 shows a four-way Venn diagram of the total 4382 DEGs detected in all shoot and root samples of PL177 and IR64 under salt+ABA treatment compared with those under only salt stress. Although largely tissue specific, the majority of these DEGs were common in both genotypes, and only 52/160 and 63/70 genes were up-regulated, and 113/114 and 137/121 genes were down-regulated exclusively by exogenous ABA in the shoots and roots of PL177/IR64, respectively.

There were 244 and 309 genes that were commonly up- and down-regulated in the shoots and 164 and 641 genes commonly up- and down-regulated in the roots of both genotypes, indicating that they were involved ABA-mediated pathways (Fig. 7). GO analysis indicated that these tissue-specific DEGs were functionally enriched in unique functional categories. The overdominant function categories for the up-regulated genes in the shoots of both genotypes included the lipid metabolic process (GO: 0006629), cellular lipid metabolic process (GO: 0044255), and fatty acid metabolic process (GO: 0006631), while the commonly down-regulated genes in shoots were those involved in the response to stress (GO: 0006950) and stimulus (GO: 0050896), and cytoplasmic vesicle (GO: 0031410), implying that ABA-mediated pathways for salt tolerance include lipid and fatty acid metabolism and cytoplasmic transport in the shoots (Supplementary Fig. S5C).

Significantly enriched genes commonly up-regulated in the roots of both genotypes included oxidoreductase activity (GO:0016491), cation binding (GO:0043169), catalytic activity (GO:0003824), metal ion binding (GO:0046872), and transferase activity (GO:0016740), while those commonly down-regulated genes in the roots of both genotypes were involved in photosynthesis (GO:0015979), photosynthetic membrane (GO:0034357), cellular metabolic process (GO:0044237), cellular biosynthetic process (GO:0044249), translation (GO:0006412), transcription regulator activity (GO:0030528), and cell membrane (GO:0005623) (Supplementary Fig. S5D).

Table 2 shows the list of 66 DEGs that are known to be functionally involved in ABA biosynthesis/signaling/response, Na^+^ and K^+^ homeostasis, and salt tolerance in plants. Of these, only three genes (protein phosphatase 2C, inwardly rectifying potassium channel, and heat-shock protein DnaJ) were up-regulated in the shoot of one or both genotypes. Thirteen of these genes were up-regulated in the roots of one or both genotypes but down-regulated in the shoots of one or both genotypes. These included three late embryogenesis abundant (LEA) group family proteins, four dehydrin family proteins, one heat-shock transcription factor and heat-shock protein 70.

**Table 2.** Relative expression levels (compared with salt stress) of 66 genes in shoots and roots of PL177 and IR64 under the salt+ABA treatment, that are known to be functionally involved in the ABA biosynthesis/signaling, and Na^+^ and K^+^ transport, and salt tolerance in plants

Gene ID	PL177 shoot	IR64 shoot	PL177 root	IR64 root	Function annotation
Os10g0541200	2.39	2.84			Protein phosphatase 2C
Os02g0245800	2.30	2.08			Inwardly rectifying potassium channel
Os03g0304500		2.25			Heat-shock protein DnaJ
Os03g0168100	0.24	0.45	2.16	2.09	Late embryogenesis abundant protein
Os08g0327700	0.17	0.35	2.03	2.23	LEA group 1 family protein
Os03g0277300	0.34		2.10	2.26	Heat-shock protein 70
Os01g0702500	0.33		2.50	2.55	Dehydrin RAB25
Os01g0705200	0.50		2.85	3.17	Late embryogenesis abundant protein
Os05g0542500			2.79	3.03	Group 3 LEA protein
Os03g0322900			3.97	4.71	Late embryogenesis abundant protein
Os11g0454000	0.34			2.49	Dehydrin family protein
Os11g0454300				2.08	Dehydrin family protein
Os01g0571300	0.39	0.42		2.35	Heat-shock transcription factor 31
Os11g0454200	0.29	0.42		2.38	Dehydrin RAB 16B
Os06g0324400	0.12	0.16		2.97	LEA group 1 family protein
Os04g0589800	0.29	0.38		2.08	LEA group 1 family protein
Os08g0544400	0.36	0.32			ABC transporter
Os07g0281800	0.40	0.33			Aldehyde oxidase-2
Os07g0154100	0.21	0.36			OsNCED3
LOC_Os09g31486	0.31	0.44			Heat-shock 70kDa protein
Os09g0526600	0.35	0.47			Heat-shock factor protein 3 (HSF 3)
Os08g0546800	0.24	0.29			Heat-shock factor RHSF2
Os05g0529700	0.47	0.50			Heat-shock protein DnaJ family protein
Os03g0266300	0.30	0.40			Heat-shock protein Hsp20
Os04g0568700	0.42	0.46			Heat stress transcription factor Spl7
Os11g0451700	0.19	0.31			Dehydrin 9
Os11g0453900	0.33	0.47			Dehydrin RAB 16D
Os09g0325700	0.38	0.34			Protein phosphatase 2C
Os06g0698300	0.30	0.39			Protein phosphatase 2C
Os02g0598500	0.45	0.46			Protein phosphatase 2C
Os05g0572700	0.19	0.24			Protein phosphatase 2C
Os04g0403600	0.42	0.43			Protein phosphatase 2C
Os07g0666900	0.41	0.43			Na/H antiporter Nhx1
Os03g0113700	0.47				Heat-shock 70kDa protein
Os10g0419300	0.43				Heat-shock factor protein 1
Os01g0840100	0.47				Heat-shock protein 70
Os05g0460000	0.49				Heat-shock protein 70
Os04g0107900	0.32				Heat-shock protein 81-1
Os06g0253100	0.49				Heat-shock protein Hsp20
Os04g0538000	0.50				Heat-shock protein STI
Os01g0225600	0.29				Late embryogenesis abundant protein
Os03g0761700		0.50			Heat-shock protein DnaJ family protein
Os04g0675400		0.49			Heat-shock protein DnaJ family protein
Os02g0575500			0.38	0.43	ABC transporter-like protein
Os01g0606900			0.17	0.13	Heat-shock protein DnaJ
Os12g0498500			0.24	0.26	Heat-shock protein DnaJ
Os03g0198300			0.47	0.49	Heat-shock protein DnaJ
Os03g0161900			0.27	0.25	Heat-shock transcription factor 21
Os09g0124100			0.19	0.20	TPR-like domain-containing protein
Os07g0171100			0.22	0.24	TPR-like domain-containing protein
Os12g0189700			0.24	0.24	TPR-like domain-containing protein
Os10g0181200			0.27	0.31	TPR-like domain-containing protein
Os01g0358300			0.33	0.39	TPR-like domain-containing protein
Os03g0336000			0.33	0.42	TPR-like domain-containing protein
Os03g0308800			0.35	0.36	TPR-like domain-containing protein
Os07g0191500			0.38	0.39	TPR-like domain-containing protein
Os04g0645100			0.39	0.47	TPR-like domain-containing protein
Os01g0510600			0.39	0.43	TPR-like domain-containing protein
Os04g0544400			0.42	0.42	TPR-like domain-containing protein
Os08g0191900			0.46	0.47	TPR-like domain-containing protein
Os05g0382200			0.22	0.31	Na+/H+ exchanging protein-like
Os04g0445000			0.41	0.42	Potassium channel SKOR
Os03g0211600			0.48		TPR-like domain-containing protein
Os02g0470000			0.48		TPR-like domain-containing protein
Os07g0287100			0.50		TPR-like domain-containing protein
Os10g0460900			0.43		TPR-like domain-containing protein

There were 27 genes that were down-regulated in the shoot of one or both genotypes. These included 12 heat-shock proteins, five protein phosphatase 2C family proteins, an ABC transporter, *Aldehyde oxidase-2*, *OsNCED3*, *Na/H antiporter Nhx1*, *Dehydrin 9* and *Dehydrin RAB 16D*, and one LEA protein. The remaining 23 genes were all down-regulated in the roots of both genotypes or in PL177, and included an ABC transporter-like protein, Na^+^/H^+^ exchanging protein-like, Potassium channel SKOR, three heat-shock DnaJ proteins, and 16 tetratricopeptide repeat-containing (TPR)-like domain-containing proteins. We noted that four genes (*Aldehyde oxidase-2*, *OsNCED3* and two ABC transporters) involved in ABA biosynthesis and transport (Hwang *et al.*, 2010; Kuromori *et al.*, 2010, Chen *et al.*, 2014) were repressed or down-regulated in the shoots or roots of both genotypes (Table 2). All the results indicated that ABA biosynthesis and transport were affected in both genotypes under salt+ABA conditions.

### Association of gene expression changes with metabolic changes under salt conditions

Table 3 shows the relative expression levels of genes involved in either biosynthesis or degradation process of those metabolites that showed strong salt-induced accumulation in the shoots and/or roots of IR64 and PL177. It appeared that strong salt-induced accumulation of three sugars (glucose, fructose, and fructofuranose) in the shoots of IR64 and PL177 could be partially attributed to reduced degradation and partially to increased biosynthesis, while strong salt-induced accumulation of proline in the shoots of both rice lines was due primarily to greatly reduced degradation. The strong salt-induced accumulation of allantoin and urea in the roots of IR64 and PL177 was inferred primarily due to reduced degradation and partially to reduced transportation (urea), because genes for their biosynthesis showed either reduced (urea) or unchanged (allantoin) expression levels when compared with the non-stress control.

**Table 3.** Coordinated changes in gene expression that led to accumulation of some key primary metabolites in shoots or roots of the salt-sensitive rice line IR64 and its salt-tolerant progeny PL177 under salt (S), salt+ABA (SA) and control (C) treatments

Organ	Metabolites	Gene codes	Gene annotation	Fold changes of metabolites				Fold changes of gene expression				Function
				PL177		IR64		PL177		IR64		
				S/C	SA/C	S/C	SA/C	S/C	SA/C	S/C	SA/C	
Shoot	Fructofuranose	LOC_Os03g28330	Sucrose synthase	12.3	27.4	6.2	58.6	1.13	1.44	0.67	1.19	Biosynthesis
		LOC_Os02g09170	Sucrose-phosphate synthase					0.74	0.63	0.82	0.57	Degradation
Shoot	Fructose	LOC_Os08g02700	Fructose-bisphospate aldolase isozyme	14.8	22.3	28.1	515.1	2.07	1.41	2.50	1.84	Biosynthesis
		LOC_Os02g48360	Pyrophosphate–fructose-6-phosphate 1-phosphotransferase					0.73	1.43	0.80	1.84	Degradation
Shoot	Glucose	LOC_Os06g14510	Glucose-9-phosphate isomerase	6.0	12.7	4.5	22.4	1.21	1.38	1.39	1.51	
		LOC_Os11g10520	UDP-glucose 8-dehydrogenase					0.84	0.73	0.50	0.56	Degradation
		LOC_Os07g13770	UDP-glucuronosyl/UDP-glucosyltransferase					1.25	1.51	1.21	1.31	Biosynthesis
Shoot	Proline	LOC_Os10g40360	Proline dehydrogenase	66.0	25.3	203.8	768.9	0.09	0.35	0.08	0.39	Degradation
Root	Urea	LOC_Os04g58390	Allantoinase	-1.5	-1.4	49.0	30.3	0.79	0.87	0.76	0.84	Biosynthesis
		LOC_Os10g42960	Urea active transporter-like protein					1.07	0.60	0.75	0.37	Transport
Root	Proline	LOC_Os10g40360	proline dehydrogenase	2.8	3.3	8.4	15.9	0.16	0.07	0.22	0.08	Degradation
Root	Allantoin	LOC_Os03g27320	Hydroxyisourate hydrolase	111.9	57.7	-1	41.8	1.03	1.16	0.93	0.99	Biosynthesis

## Discussion

### Physiological mechanisms of salt tolerance of PL177

Because of its wide adaptability, high yield, and desirable quality, IR64 has been widely grown in south and south-east Asia for several decades, even though it does not have good tolerance to salt and drought. Previously, we were able to develop many pyramiding lines with greatly improved salt tolerance including PL177. In this study, we demonstrated that PL177 was very similar to IR64 phenotypically under non-stress conditions but with significantly improved seedling salt tolerance, which was reflected by much less sodium accumulation in both roots and shoots and less shoot damage than IR64, apparently resulting from its lower Na^+^ concentration and higher K^+^ concentration in both tissues and less Na^+^ translocation from the roots to the shoots under salt stress. All these results suggested that the physiological mechanisms of salt tolerance in PL177 are salt exclusion in its roots and salt compartmentation in shoots, which results in its improved capacity to maintain a lower Na^+^/K^+^ ratio in shoots and a relatively higher growth rate under salt.

### Metabolic responses underlying salt tolerance in rice

Consistent with previous findings (Sanchez *et al.*, 2008; El-Samad *et al.*, 2010), our results revealed two important aspects of the metabolic responses of rice to salt stress. First, there were general increases of many primary metabolites in both rice genotypes in response to salt stress, and the salt- and/or salt+ABA-induced metabolic responses were much more pronounced in the shoots than in the roots of both genotypes, which were partially ABA mediated and partially ABA independent. Secondly, strong salt-induced accumulation was observed for only a few primary metabolites, which were highly organ specific, with several sugars (glucose, raffinose, fructose, fructofuranose, and tryptophan) plus proline accumulating strongly in the shoots of both genotypes, while three primary products in the N metabolism (allantoin, urea, and glutamine) accumulated strongly in the roots of both genotypes. Physiologically, many of these metabolites can act as osmolytes in the cytoplasm under salt stress, resulting in increased osmotic potential of the cytoplasm in shoot and/or root cells to maintain appropriate cell water status and protect proteins from salt-induced dissociation, while accumulated organic acids and small molecular components might be involved in the compensation of ionic imbalance and carbon metabolism (López-Bucio *et al.*, 2000; Szabados and Savouré, 2010). We further noted that the greatest genotypic differences in salt-induced accumulation were proline and fructose in shoots and allantoin in roots, in which the proline and fructose levels in shoots of PL177 were 5.2-fold and 8.8-fold higher than those in IR64, while the allantoin level in the roots of PL177 was 42.6-fold higher than in IR64 roots only under salt stress. This strongly suggests that salt-induced accumulation of these metabolites may contribute directly to the improved salt tolerance of PL177. This differentiated accumulation of sugars in shoots and N metabolites in roots of rice under salt stress is energetically more efficient, as the biosynthesis and degradation of sugars occur in shoots, and those metabolites in the N metabolic pathways (allantoin and urea) occur in roots (Fig. 4). In this respect, our results appear to support the notion that stress-induced accumulation of specific metabolites contributes directly to salt tolerance of rice. It should be pointed out that the primary metabolites measured in this study represented only a portion of the metabolome responsive to salt, and other metabolites may also contribute to the salt tolerance of rice. For example, lipids and fatty acids may also contribute to the salt tolerance of rice, as genes involved in lipid and fatty acid metabolic pathways were significantly up-regulated by salt based on the transcriptome data discussed below.

### Transcriptomic responses underlying salt tolerance in rice

Extensive transcriptomic studies have revealed that plants can adapt to different environmental stresses by widely reshaping their transcriptome in an organ-specific manner (Wang *et al.*, 2011; Brenner and Schmülling, 2012; Begara-Morales *et al.*, 2014). Consistent with previous studies, large numbers of organ-specific DEGs were detected in both rice genotypes in their responses to salt and salt+ABA. As expected, most up-regulated genes in the shoots of both genotypes included lipid and fatty acid metabolic processes, while the commonly down-regulated genes in shoots were involved in the stress response and cytoplasmic vesicles. In contrast, DEGs commonly up-regulated in the roots of both genotypes were involved in oxidoreductase activity, cation binding, catalytic activity, metal ion binding, and transferase activity, while the commonly down-regulated genes in roots of both genotypes functioned in photosynthesis, photosynthetic membranes, cellular metabolic processes, cellular biosynthetic processes, translation, transcription regulator activity, and the cell membrane.

ABA is known to have massive influences on rice responses to salt at the phenotypic, metabolomics, and transcription levels (Du *et al.*, 2013; Gurmani *et al.*, 2013; Sripinyowanich *et al.*, 2013). In this study, exogenous ABA was able to alleviate the salt stress effect on the salt-sensitive line IR64, indicating that the ABA-mediated system for salt tolerance is at least partially impaired in IR64. Indeed, exogenous ABA greatly up-regulated the genes involved in the lipid and fatty acid metabolisms and cytoplasmic transport in shoots of both genotypes under salt stress, suggesting that enhanced lipid metabolism might also contribute to enhanced salt tolerance of rice. Down-regulated genes specifically in the roots or shoots of both genotypes by ABA included most heat-shock proteins, heat-shock transcription factors, TPR proteins, protein phosphatase 2Cs, a Na^+^/H^+^ antiporter, a Na^+^/H^+^ exchanging protein, and a potassium channel SKOR that are known to be involved in osmotic stress regulation, Na^+^ and K^+^ homeostasis, and stress resistance in ABA signaling pathways (Table 2) (Qiu *et al.*, 2002; Pilot *et al.*, 2003; Rosado *et al.*, 2006; Schapire *et al.*, 2006; Zou *et al.*, 2009; Fukuda *et al.*, 2010; Bassil *et al.*, 2011; Ye *et al.*, 2012). Furthermore, ABA-mediated repression of genes for degradation of key primary metabolites such as proline and allantoin was responsible for their salt-induced accumulation in rice shoots and/or roots observed in this study. We found that those genes for ABA biosynthesis in roots under salt+ABA treatment were significantly down-regulated and so were for those for ABA transport in the shoots and roots, suggesting that genes involved in endogenous ABA signaling are feedback regulated by exogenous ABA.

The transcriptomic differences between IR64 and PL177 provided us with a unique opportunity to gain insights into key candidate genes and the molecular mechanisms underlying salt tolerance in rice. Many genes specifically up-regulated in PL177 under salt conditions are known to be functionally involved in salt tolerance, including *Calcinerin B*, *Potassium transporter 7*, and *PDR-like ABC transporter* in calcium signaling, potassium influx, and detoxification (Gu *et al.*, 2008; Yang *et al.*, 2009; Nuruzzaman *et al.*, 2014). In particular, genes involved in the monocarboxylic acid metabolic process, and oxidoreductase and monooxygenase activities were specifically up-regulated in PL177 roots, implying enhanced oxidation–reduction reactions in the roots of PL177. Furthermore, genes involved in cytoplasmic vesicles, hydrolase activity, the polysaccharide metabolism process, hydrolyzing *O*-glycosyl compounds, and the extracellular region, and gene families including glycoside hydrolases and lipid transfer proteins related to cell-wall remodeling (Carvalho and Gomes, 2007; Minic, 2008) were specifically up-regulated in PL177 shoots, suggesting that sequestration by vacuoles, detoxification, and cell-wall remodeling are key mechanisms in shoots contributing to salt tolerance in rice. Notably, some genes related to oxidoreductase activity and photosynthesis were enriched in the roots of PL177. Some early studies reported that genes involved in photosynthesis are normally down-regulated in the roots of rice plants under low phosphorus stress (Li *et al.*, 2010) and low nitrogen stress (Lian *et al.*, 2006), but are significantly induced under dehydration stress (Minh-Thu *et al.*, 2013). The biological relevance of salt-induced photosynthesis gene expression in roots needs to be further elucidated. Finally, by mapping DEGs specifically up-regulated in PL177 onto the donor segments in PL177, we were able to shortlist only 43 candidate genes for salt tolerance in PL177 introgressed from the donors, including an ABA-responsive element binding protein, a Zn-finger, a RING domain-containing protein, Annexin p33, and a receptor kinase-like protein. Transformation experiments by genetic complementation or knockout to verify the functionality of these genes on salt tolerance are in progress.

## Supplementary data

Supplementary data are available at *JXB* online.

**Supplementary Fig. S1.** The BC breeding and intercross procedures for developing salt-tolerant pyramiding lines.

**Supplementary Fig. S2.** Genetic composition of PL177 at six genomic regions (loci).

**Supplementary Fig. S3.** Schedule of salt and salt+ABA treatments for PL177 and IR64.

**Supplementary Fig. S4.** Validation of the expression of 10 selected genes by qRT-PCR.

**Supplementary Fig. S5.** Metabolic overview of DEGs in PL177 and IR64 under salt and salt+ABA conditions.

**Supplementary Table S1.** The modified standard evaluation system (SES) of visual salt injury at the seedling stage of rice.

**Supplementary Table S2.** The mean values of 88 primary metabolites from shoots and roots of IR64 and PL177 measured at control (C), salt (S), and salt+ABA (SA) conditions.

**Supplementary Table S3.** Morphological and yield trait performances of IR64 and PL177 measured under control, salt, and salt+ABA conditions.

**Supplementary Table S4.** Shoot and root sodium and potassium concentrations in IR64 and PL177 under salt and salt+ABA treatment conditions.

**Supplementary Table S5.** Summary of the fold changes of 88 primary metabolites in two genotypes (PL177 and IR64) in response to salt stress and salt+ABA treatments at the seedling stage.

**Supplementary Table S6.** ANOVA results for 88 primary metabolites in shoots (A) and roots(B) of two rice genotypes (G), IR64 and PL177, measured under three different treatments (Trt), control, salt, and salt+ABA (SA).

**Supplementary Table S7.** The loadings (contributions) of each of the 88 metabolites to the first three principal components (PCs).

**Supplementary Table S8.** Significant fold changes (in concentrations) of some metabolites in two rice genotypes (IR64 and PL177) in response to the salt and salt+ABA (SA) treatments at the seedling stage.

**Supplementary Table S9.** List of primers of genes for the quantitative RT-PCR.

**Supplementary Table S10.** Common DEGs detected in both shoots and roots of both genotypes under salt stress.

**Supplementary Table S11.** GO enrichment analysis of the PL177-specific DEGs under salt stress.

**Supplementary Table S12.** List of genes that were exclusively expressed in PL177 that were all mapped on the introgressed chromosome regions of PL177.

Supplementary Data

## References

[CIT0001] Ashrafa M Foolad MR 2007 Roles of glycine betaine and proline in improving plant abiotic stress resistance. Environmental and Experimental Botany 59, 206–216.

[CIT0002] Bassil E Tajima H Liang YC Ohto MA Ushijima K Nakano R Esumi T Coku A Belmonte M Blumwald E 2011 The Arabidopsis Na^+^/H^+^ antiporters NHX1 and NHX2 control vacuolar pH and K^+^ homeostasis to regulate growth, flower development, and reproduction. The Plant Cell 23, 3482–3497. 21954467 10.1105/tpc.111.089581 PMC3203450

[CIT0003] Begara-Morales JC Sánchez-Calvo B Luque F Leyva-Pérez MO Leterrier M Corpas FJ Barroso JB 2014 Differential transcriptomic analysis by RNA-Seq of GSNO-responsive genes between Arabidopsis roots and leaves. Plant and Cell Physiology 55, 1080–1095. 24599390 10.1093/pcp/pcu044

[CIT0004] Brenner WG Schmülling T 2012 Transcript profiling of cytokinin action in Arabidopsis roots and shoots discovers largely similar but also organ-specific responses. BMC Plant Biology 12, 112. 22824128 10.1186/1471-2229-12-112 PMC3519560

[CIT0005] Carvalho Ade O Gomes VM 2007 Role of plant lipid transfer proteins in plant cell physiology—a concise review. Peptides 28, 1144–1153. 17418913 10.1016/j.peptides.2007.03.004

[CIT0006] Chen QF Ya HY Feng YR Jiao Z 2014 Expression of the key genes involved in ABA biosynthesis in rice implanted by ion beam. Applied Biochemistry and Biotechnology 173, 239–247. 24634194 10.1007/s12010-014-0837-y

[CIT0007] Cotsaftis O Plett D Johnson AA Walia H Wilson C Ismail AM Close TJ Tester M Baumann U 2011 Root-specific transcript profiling of contrasting rice genotypes in response to salinity stress. Molecular Plant 4, 25–41. 20924028 10.1093/mp/ssq056

[CIT0008] Du YL Wang ZY Fan JW Turner NC He J Wang T Li FM 2013 Exogenous abscisic acid reduces water loss and improves antioxidant defence, desiccation tolerance and transpiration efficiency in two spring wheat cultivars subjected to a soil water deficit. Functional Plant Biology 40, 494–506. 10.1071/FP12250 32481126

[CIT0009] Du Z Zhou X Ling Y Zhang ZH Su Z 2010 agriGO: a GO analysis toolkit for the agricultural community. Nucleic Acids Research 38, W64–W70. 20435677 10.1093/nar/gkq310 PMC2896167

[CIT0010] El-Samad HM Abd Shaddad MAK Barakat N 2010 The role of amino acids in improvement in salt tolerance of crop plants. Journal of Stress Physiology Biochemistry 6, 25–37.

[CIT0011] Fukuda A Nakamura A Hara N Toki S Tanaka Y 2010 Molecular and functional analyses of rice NHX-type Na^+^/H^+^ antiporter genes. Planta 233, 175–188. 20963607 10.1007/s00425-010-1289-4

[CIT0012] Gregorio GB Senadhira D Mendoza RD 1997 *Screening rice for salinity tolerance. IRRI Discussion Paper Series Number 22* . International Rice Research Institute: Manila, The Philippines.

[CIT0013] Gurmani AR Bano A Najeeb U Zhang JL Khan SU Flowers TJ 2013 Exogenously applied silicate and abscisic acid ameliorates the growth of salinity stressed wheat (*Triticum aestivum* L) seedling through Na^+^ exclusion. Australian Journal of Crop Science 7, 1123–1130.

[CIT0014] Gu Z Ma B Jiang Y Chen Z Su X Zhang H 2008 Expression analysis of the calcineurin B-like gene family in rice (*Oryza sativa* L.) under environmental stresses. Gene 415, 1–12. 18395997 10.1016/j.gene.2008.02.011

[CIT0015] Hwang SG Chen HC Huang WY Chu YC Shii CT Cheng WH 2010 Ectopic expression of rice *OsNCED3* in Arabidopsis increases ABA level and alters leaf morphology. Plant Science 178, 12–22.

[CIT0016] Kawasaki S Borchert C Deyholos M Wang H Brazille S Kawai K Galbraith D Bohnert HJ 2001 Gene expression profiles during the initial phase of salt stress in rice. The Plant Cell 13, 889–905. 11283343 10.1105/tpc.13.4.889 PMC135538

[CIT0017] Khadri M Tejera NA Lluch C 2007 Sodium chloride-ABA interaction in two common bean (*Phaseolus vulgaris*) cultivars differing in salinity tolerance. Environmental and Experimental Botany 60, 211–218.

[CIT0018] Kreps JA Wu Y Chang HS Zhu T Wang X Harper JF 2002 Transcriptome changes for Arabidopsis in response to salt, osmotic, and cold stress. Plant Physiology 130, 2129–2141. 12481097 10.1104/pp.008532 PMC166725

[CIT0019] Kumar K Kumar M Kim SR Ryu H Cho YG 2013 Insights into genomics of salt stress response in rice. Rice 6, 27. 24280112 10.1186/1939-8433-6-27 PMC4883734

[CIT0020] Kumari S Sabharwal VP Kushwaha HR Sopory SK Singla-Pareek SL Pareek A 2009 Transcriptome map for seedling stage specific salinity stress response indicates a specific set of genes as candidate for saline tolerance in *Oryza sativa* L. Functional Integrative Genomics 9, 109–123. 18594887 10.1007/s10142-008-0088-5

[CIT0021] Kuromori T Miyaji T Yabuuchi H Shimizu H Sugimoto E Kamiya A Moriyama Y Shinozaki K 2010 ABC transporter *AtABCG25* is involved in abscisic acid transport and responses. Proceedings of the National Academy of Sciences, USA 107, 2361–2366. 10.1073/pnas.0912516107 PMC2836683 20133881

[CIT0022] Larbi A Mekliche A 2004 Relative water content (RWC) and leaf senescence as screening tools for drought tolerance in wheat. In Cantero-Martínez C Gabiña D, eds. *Mediterranean rainfed agriculture: strategies for sustainability* . CIHEAM: Zaragoza, pp. 193–196.

[CIT0023] Li HW Zang BS Deng XW Wang XP 2011 Overexpression of the trehalose-6-phosphate synthase gene *OsTPS1* enhances abiotic stress tolerance in rice. Planta 234, 1007–1018. 21698458 10.1007/s00425-011-1458-0

[CIT0024] Li LH Qiu XH Li XH Wang SP Zhang QF Lian XM 2010 Transcriptomic analysis of rice responses to low phosphorus stress. Chinese Science Bulletin 55, 251–258.

[CIT0025] Li ZK Xu JL 2007 Breeding for drought and salt tolerant rice (*Oryza Sativa* L.): progress and perspectives. In: Jenks MA Hasegawa PM Jain SM, eds. *Advances in molecular breeding toward drought and salt tolerant crops* , pp. 531–564.

[CIT0026] Lian X Wang S Zhang J Feng Q Zhang L Fan D Li X Yuan D Han B Zhang Q 2006 Expression profiles of 10,422 genes at early stage of low nitrogen stress in rice assayed using a cDNA microarray. Plant Molecular Biology 60, 617–631. 16649102 10.1007/s11103-005-5441-7

[CIT0027] Lin HX Zhu MZ Yano MJ Gao P Liang ZW Su WA Hu XH Ren ZH Chao DY 2004 QTLs for Na^+^ and K^+^ uptake of the shoots and roots controlling rice salt tolerance. Theoretical and Applied Genetics 108, 253–260. 14513218 10.1007/s00122-003-1421-y

[CIT0028] López-Bucio J Nieto-Jacobo MF Ramírez-Rodríguez V Herrera-Estrella L 2000 Organic acid metabolism in plants: from adaptive physiology to transgenic varieties for cultivation in extreme soils. Plant Science 160, 1–13. 11164572 10.1016/s0168-9452(00)00347-2

[CIT0029] Lutts S Kinet JM Bouharmont J 1995 Changes in plant response to NaCl during development of rice (*Oryza sativa* L.) varieties differing in salinity resistance. Journal of Experimental Botany 46, 1843–1852.

[CIT0030] Minh-Thu PT Hwang DJ Jeon JS Nahm BH Kim YK 2013 Transcriptome analysis of leaf and root of rice seedling to acute dehydration. Rice 6, 38. 24341907 10.1186/1939-8433-6-38 PMC3878681

[CIT0031] Minic Z 2008 Physiological roles of plant glycoside hydrolases. Planta 227, 723–740. 18046575 10.1007/s00425-007-0668-y

[CIT0032] Nuruzzaman M Zhang R Cao HZ Luo ZY 2014 Plant pleiotropic drug resistance transporters: transport mechanism, gene expression, and function. Journal of Integrative Plant Biology 56, 729–740. 24645852 10.1111/jipb.12196

[CIT0033] Ondrasek G Rengel Z Veres S 2011 Soil salinisation and salt stress in crop production. In: Shanker AK Venkateswarlu B eds. *Abiotic stress in plants: mechanisms and adaptations* . Rijeka, Croatia: InTech, pp. 171–190.

[CIT0034] Pilot G Gaymard F Mouline K Chérel I Sentenac H 2003 Regulated expression of Arabidopsis shaker K^+^ channel genes involved in K^+^ uptake and distribution in the plant. Plant Molecular Biology 51, 773–787. 12678562 10.1023/a:1022597102282

[CIT0035] Qiu QS Guo Y Dietrich MA Schumaker KS Zhu JK 2002 Regulation of SOS1, a plasma membrane Na^+^/H^+^ exchanger in *Arabidopsis thaliana*, by SOS2 and SOS3. Proceedings of the National Academy of Sciences, USA 99, 8436–8441. 10.1073/pnas.122224699 PMC123085 12034882

[CIT0036] Ren ZH Gao JP Li LG Cai XL Huang W Chao DY Zhu MZ Wang ZY Luan S Lin HX 2005 A rice quantitative trait locus for salt tolerance encodes a sodium transporter. Nature Genetics 37, 1141–1146. 16155566 10.1038/ng1643

[CIT0037] Rosado A Schapire AL Bressan RA Harfouche AL Hasegawa PM Valpuesta V Botella MA 2006 The Arabidopsis tetratricopeptide repeat-containing protein TTL1 is required for osmotic stress responses and abscisic acid sensitivity. Plant Physiology 142, 1113–1126. 16998088 10.1104/pp.106.085191 PMC1630727

[CIT0038] Sanchez DH Siahpoosh MR Roessner U Udvardi M Kopka J 2008 Plant metabolomics reveals conserved and divergent metabolic responses to salinity. Physiology Plant 132, 209–219. 10.1111/j.1399-3054.2007.00993.x 18251862

[CIT0039] Schapire AL Valpuesta V Botella MA 2006 TPR proteins in plant hormone signaling. Plant Signal and Behavior 1, 229–230. 10.4161/psb.1.5.3491 PMC2634123 19704665

[CIT0040] Singh RK Gregorio GB Jain RK 2007 QTL mapping for salinity tolerance in rice. Physiology and Molecular Biology of Plants 13, 87–99.

[CIT0041] Sripinyowanich S Klomsakul P Boonburapong B Bangyeekhun T Asami T Gu HY Buaboocha T Chadchawan S 2013 Exogenous ABA induces salt tolerance in indica rice (*Oryza sativa* L.): the role of *OsP5CS1* and *OsP5CR* gene expression during salt stress. Environmental and Experimental Botany 86, 94–105.

[CIT0042] Szabados L Savouré A 2010 Proline: a multifunctional amino acid. Trends in Plant Science 15, 89–97. 20036181 10.1016/j.tplants.2009.11.009

[CIT0043] Thomson MJ de Ocampo M Egdane J 2010 Characterizing the Saltol quantitative trait locus for salinity tolerance in rice. Rice 3, 148–160.

[CIT0044] Thimm O Bläsing O Gibon Y Nagel A Meyer S Krüger P Selbig J Müller LA Rhee SY Stitt M 2004 Mapman: a user-driven tool to display genomics data sets onto diagrams of metabolic pathways and other biological processes. The Plant Journal 37, 914–939. 14996223 10.1111/j.1365-313x.2004.02016.x

[CIT0045] Walia H Wilson C Zeng L Ismail AM Condamine P Close TJ 2007 Genome-wide transcriptional analysis of salinity stressed *japonica* and *indica* rice genotypes during panicle initiation stage. Plant Molecular Biology 63, 609–623. 17160619 10.1007/s11103-006-9112-0 PMC1805040

[CIT0046] Wang D Pan Y Zhao X Zhu L Fu B Li Z 2011 Genome-wide temporal-spatial gene expression profiling of drought responsiveness in rice. BMC Genomics 12, 149. 21406116 10.1186/1471-2164-12-149 PMC3070656

[CIT0047] Wang Z Chen Z Cheng J Lai Y Wang J Bao Y Huang J Zhang H 2013 QTL analysis of Na^+^ and K^+^ concentrations in roots and shoots under different levels of NaCl stress in rice (*Oryza sativa* L.). PLoS One 7, e51202. 23236455 10.1371/journal.pone.0051202 PMC3516561

[CIT0048] Yang Z Gao Q Sun C Li W Gu S Xu C 2009 Molecular evolution and functional divergence of HAK potassium transporter gene family in rice (*Oryza sativa* L.). Journal of Genetics and Genomics 36, 161–172. 19302972 10.1016/S1673-8527(08)60103-4

[CIT0049] Ye SF Yu SW Shu SB Wu JH Wu AZ Luo LJ 2012 Expression profile analysis of 9 heat shock protein genes throughout the life cycle and under abiotic stress in rice. Chinese Science Bulletin 57, 336–343.

[CIT0050] Yoshida S Forna DA Cock JH Gomez KA 1976 *Laboratory manual for physiological studies of rice* . Los Baños, The Philippines: International Rice Research Institute.

[CIT0051] Zang JP Sun Y Wang Y 2008 Dissection of genetic overlap of salt tolerance QTLs at the seedling and tillering stages using backcross introgression lines in rice. Science China Series C (Life Sciences) 51, 583–591. 10.1007/s11427-008-0081-1 18622741

[CIT0052] Zhao XQ Wang WS Zhang F Zhang T Zhao W Fu BY Li ZK 2013 Temporal profiling of primary metabolites under chilling stress and its association with seedling chilling tolerance of rice (*Oryza sativa* L.). Rice 6, 23. 24280004 10.1186/1939-8433-6-23 PMC4883686

[CIT0053] Zou J Liu A Chen X Zhou X Gao G Wang W Zhang X 2009 Expression analysis of nine rice heat shock protein genes under abiotic stresses and ABA treatment. Journal of Plant Physiology 166, 851–861. 19135278 10.1016/j.jplph.2008.11.007

