# Supplementary material for: Complex molecular mechanisms underlying seedling salt tolerance in rice revealed by comparative transcriptome and metabolomic profiling
Source: J Exp Bot. 2015 Oct 27;67(1):405–19. doi: 10.1093/jxb/erv476 (PMC4682442; doi:10.1093/jxb/erv476)
Supplement: Supplementary Data [file supp_67_1_405__index.html]

Complex molecular mechanisms underlying seedling salt tolerance in rice revealed by comparative transcriptome and metabolomic profiling — Complex molecular mechanisms underlying seedling salt tolerance in rice revealed by comparative transcriptome and metabolomic profiling — Supplementary Data 

# Complex molecular mechanisms underlying seedling salt tolerance in rice revealed by comparative transcriptome and metabolomic profiling

## Supplementary Data

Data files

- Supplementary Data - Supplementary Data
