# Supplementary material for: Complex molecular mechanisms underlying seedling salt tolerance in rice revealed by comparative transcriptome and metabolomic profiling
Source: J Exp Bot. 2015 Oct 27;67(1):405–19. doi: 10.1093/jxb/erv476 (PMC4682442; doi:10.1093/jxb/erv476)
Supplement: Supplementary Data [file supp_erv476_Supplementary_Figures_S1_S5_Tables_S1_S12.pdf]

Complex Molecular Mechanisms underlying Seedling Salt Tolerance in Rice (Oryza sativa L.) Revealed by Comparative Transcriptome and Metabolomic Profiling

Wensheng Wang, Xiuqin Zhao, Min Li, Liyu Huang, Jianlong Xu, Fan Zhang, Yanru Cui, Binying Fu, and Zhikang Li

Supplemental Files

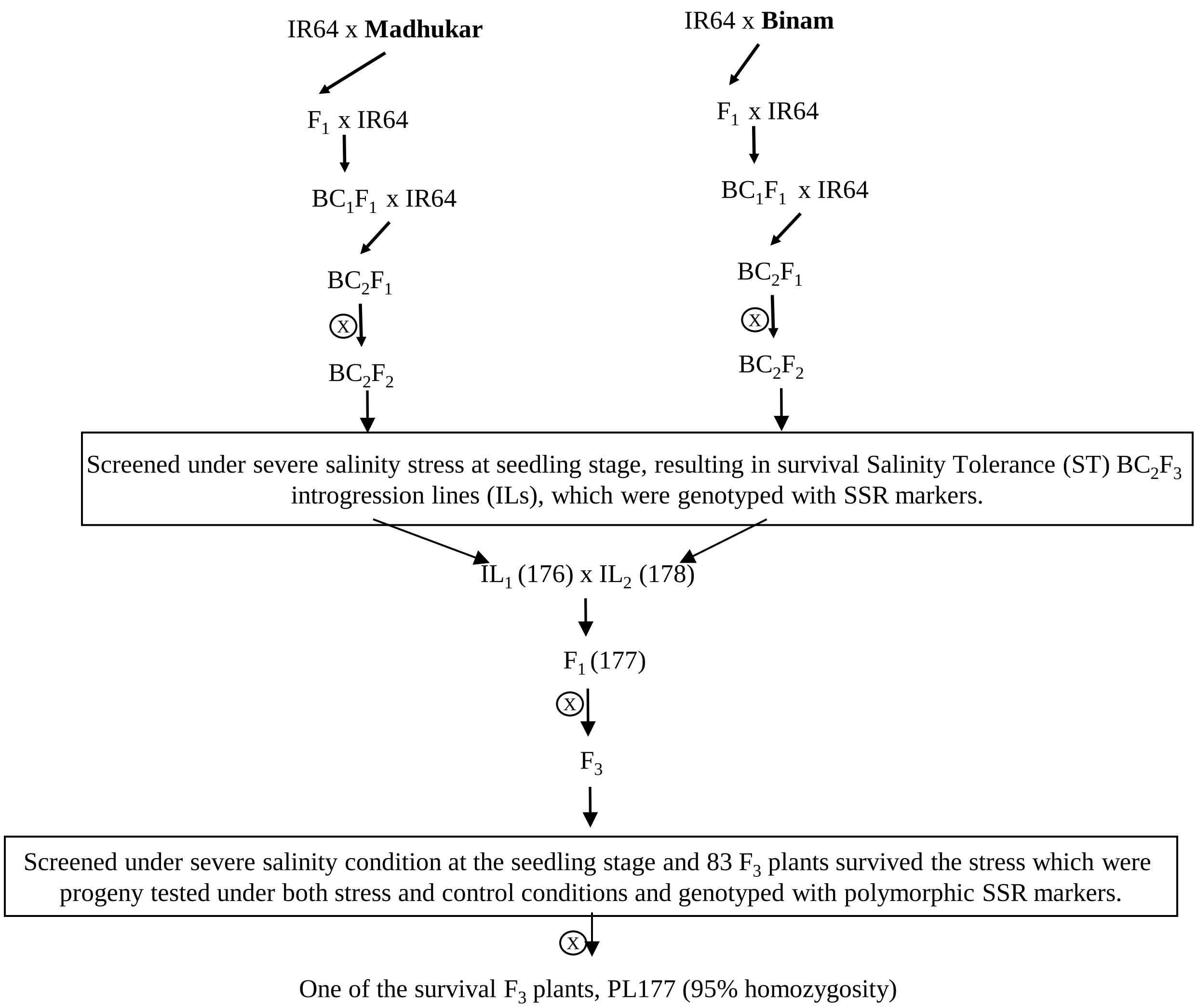

Figure S1. The BC breeding and intercross procedures for developing salinity tolerant introgression lines (176 and 178) and the pyramiding line PL177 using IR64 (the recipient) and 2 donors, Madhukar and Binam.

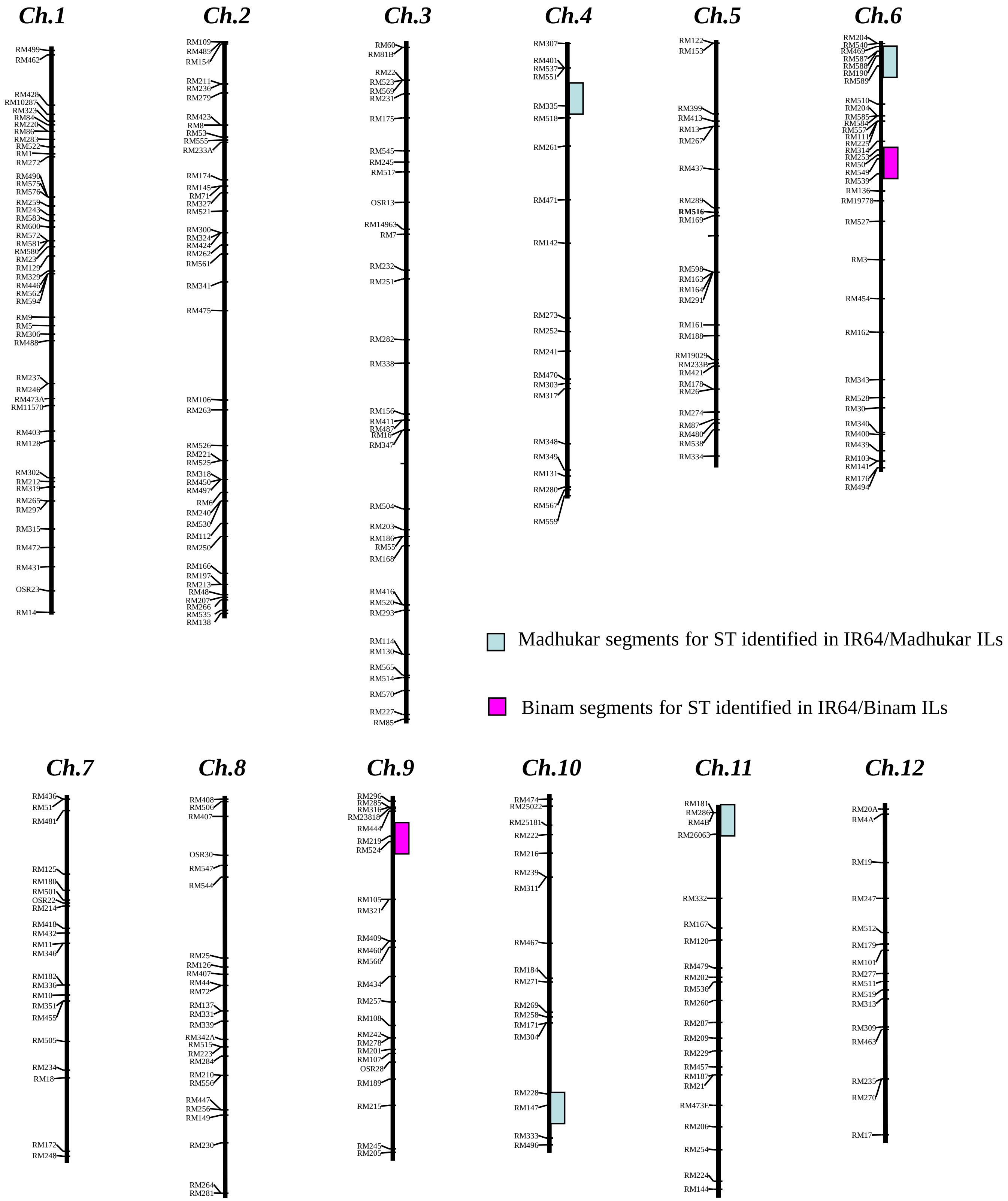

Figure S2. Genetic composition of PL177 at 6 genomic regions (loci) introgressed and pyramided from two different donors, Madhukar and Binam, in the IR64 genetic background based on SSR markers. Ch., chromosome.

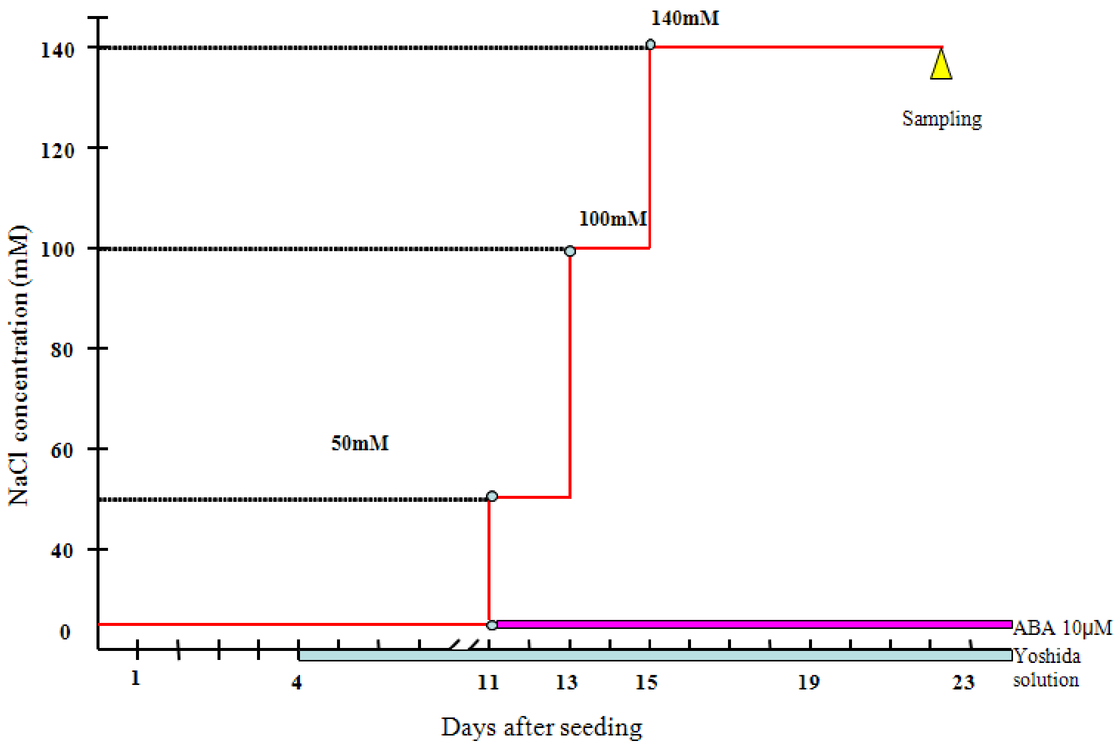

Figure S3. Schedule of salt and salt+ABA treatments for PL177 and IR64

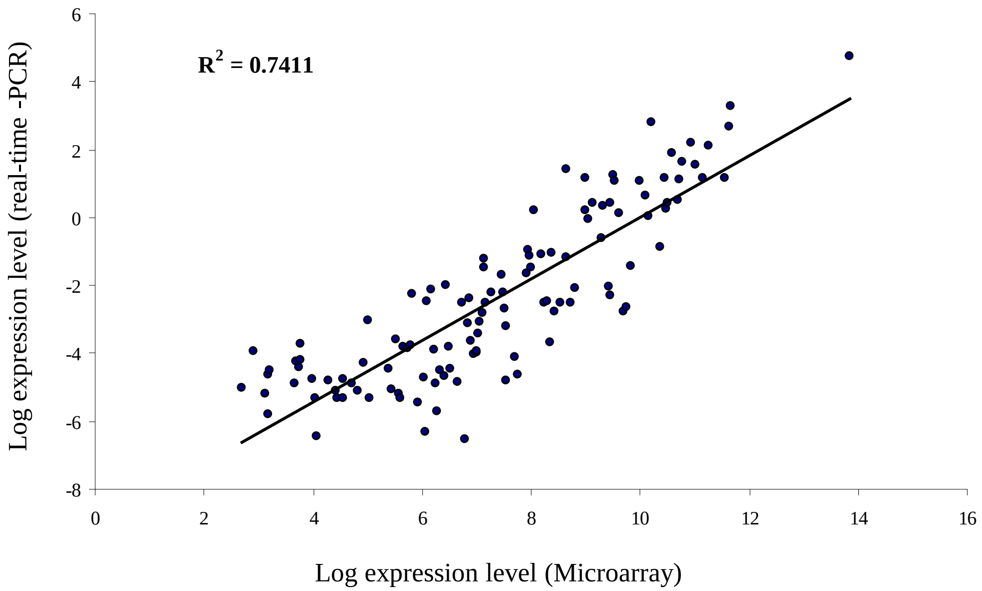

Figure S4. Validation of the expression of 10 selected genes by qRT-PCR. Correlation analysis shows a good agreement between microarray and qRT-RCR experiments. The gene expression values were transformed to log_2 scale. The microarray data log_2-value (*X*-axis) were plotted against the real-time RT-PCR log_2-value (*Y*-axis).

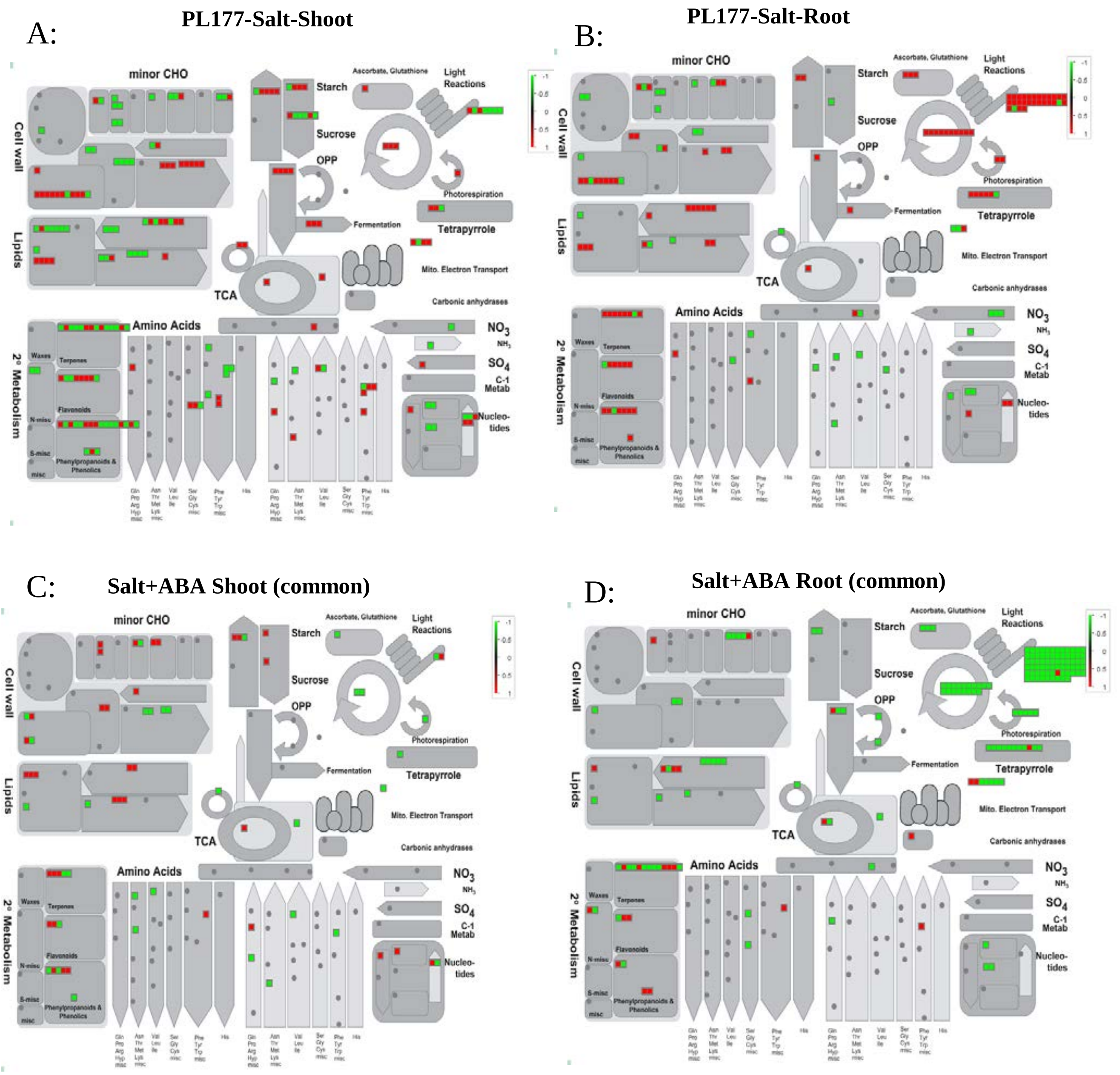

Figure S5. Metabolic overview of DEGs in PL177 and IR64 under salt and salt+ABA conditions

Table S1. The modified standard evaluation system (SES) of visual salt injury at the seedling stage of rice

Score	Observation	Tolerance
1	Normal growth, no leaf symptoms	High tolerance
3	Nearly normal growth, but leaf tips or few leaves whitish and rolled	Tolerance
5	Growth severely retarded; most leaves rolled; only a few are elongating	Moderately tolerant
7	Complete cessation of growth; most leaves dry; some plants dying	Susceptible
9	Almost all plants dead or dying	Highly susceptible

Table S2. The mean values of 88 primary metabolites from shoots and roots of IR64 and PL177 measured at control (C), salt (S) and salt plus ABA (SA) conditions

	Tissue	Shoots						Roots					
	Treatment	C		S		SA		C		S		SA	
	Genotype	PL177	IR64	PL177	IR64	PL177	IR64	PL177	IR64	PL177	IR64	PL177	IR64
Amino acids	4-Amino- Butyric Acid	16.25±1.26	10.10±0.50	70.90±7.25	55.86±13.33	69.50±14.62	51.91±6.58	2.85±0.46	2.50±0.01	3.23±1.29	3.80±1.16	3.07±0.45	4.21±0.88
	Alanine	18.91±3.44	11.89±0.95	67.98±19.30	37.57±4.99	51.81±7.67	47.69±5.72	1.53±0.54	1.56±0.46	1.20±0.14	1.73±0.14	1.11±0.14	3.08±1.41
	Arginine	24.08±1.57	27.09±3.23	65.96±21.30	92.21±13.26	87.34±6.20	62.39±6.08	1.86±0.22	2.85±0.27	4.84±0.25	4.97±1.23	7.84±1.35	15.00±1.47
	Asparagine	66.69±3.87	7.21±1.91	125.63±7.70	12.18±1.13	57.64±14.80	131.18±15.01	0.16±0.02	0.29±0.08	1.25±0.03	0.65±0.19	1.65±0.73	4.18±0.98
	Aspartic Acid	47.11±7.88	52.80±8.08	78.08±1.44	100.18±3.48	97.73±10.98	94.48±4.27	6.31±0.45	7.51±1.54	9.43±0.17	6.65±1.83	7.37±1.23	11.58±1.75
	Cytosine	24.69±2.41	22.47±7.07	55.52±13.59	45.33±5.71	49.99±3.47	40.74±4.30	4.25±0.51	3.55±0.37	4.10±1.39	4.45±0.87	3.82±0.74	4.18±1.01
	Glutamine	23.27±2.03	40.39±5.65	109.48±1.67	537.24±140.34	147.32±12.30	104.43±28.35	1.55±0.46	2.23±0.09	4.16±0.66	2.65±0.29	4.99±1.28	17.68±1.41
	Glycine	13.72±7.50	8.33±1.65	57.25±10.34	45.17±8.59	48.79±5.06	44.61±6.90	4.47±0.45	5.12±2.77	3.49±0.35	5.17±2.04	3.79±0.72	10.19±0.43
	Isoleucine	28.89±3.41	22.04±6.33	127.33±10.18	112.90±19.08	138.98±31.50	108.26±13.81	2.65±0.18	1.53±0.09	3.41±0.21	4.40±0.93	6.48±1.24	9.82±0.78
	Leucine	31.21±6.46	19.78±2.16	126.50±2.43	105.42±19.44	222.81±62.08	90.26±9.14	2.89±0.33	2.84±0.44	3.88±0.83	3.92±0.63	4.36±0.41	5.67±0.26
	Methionine	30.19±4.33	27.16±4.25	30.38±1.84	33.70±1.44	33.11±3.21	27.05±3.82	9.90±1.16	10.83±1.54	11.21±0.87	11.09±1.06	9.85±0.87	12.28±1.31
	Phenylalanine	53.42±9.85	27.34±9.56	126.03±0.34	107.27±33.25	87.01±2.60	96.82±42.00	4.47±0.43	4.09±0.84	6.88±2.10	10.64±3.54	21.97±2.82	19.08±4.99
	Proline	9.70±2.36	0.61±0.07	640.32±120.97	124.31±36.49	245.62±12.69	469.02±74.53	0.56±0.52	0.08±0.01	1.56±0.24	0.63±0.08	1.84±0.27	1.19±0.25
	Serine	28.93±3.22	24.00±2.57	119.32±37.93	114.67±6.91	109.68±19.41	112.38±12.71	5.29±0.55	6.13±0.19	5.89±0.76	4.69±1.13	3.79±0.55	4.84±0.78
	Threonine	34.55±5.28	28.26±3.33	107.17±8.68	95.35±15.18	110.25±13.67	96.35±10.56	4.32±1.02	4.71±0.02	7.51±2.35	6.32±1.65	7.72±1.26	10.34±2.18
	Tryptophan	25.99±5.48	29.03±6.53	178.64±65.28	268.12±76.37	396.65±37.42	160.33±34.88	3.87±1.30	7.09±1.09	9.54±1.32	13.85±4.47	11.06±1.52	18.51±6.00
	Tyramine	23.91±15.47	9.99±1.91	31.79±8.55	33.04±7.39	33.51±4.16	32.88±4.92	12.23±2.20	9.51±0.15	9.26±0.24	7.11±0.67	15.66±2.16	19.33±3.41
	Tyrosine	13.47±0.30	12.33±2.68	52.79±5.34	57.70±5.86	57.95±10.75	50.07±7.76	1.96±0.54	2.49±0.10	3.47±0.73	3.83±1.01	3.83±0.53	4.54±0.77
	Valine	27.08±3.85	23.82±6.08	128.11±5.35	120.73±20.64	122.46±12.41	104.63±12.26	3.32±0.77	3.56±0.05	5.39±1.13	5.57±1.39	6.63±0.58	10.83±2.30

	1-Ethylglucopyranoside	50.09±21.43	32.61±21.57	16.18±16.12	5.67±1.13	33.54±24.89	14.97±11.52	1.65±0.50	4.20±0.33	0.87±0.44	1.44±0.89	3.50±0.68	2.96±0.38
	D-Xylofuranose	38.57±11.82	47.21±7.16	41.39±4.43	54.99±7.12	43.38±5.48	39.54±1.77	3.85±0.77	4.82±0.60	2.42±0.15	2.95±0.16	2.19±0.27	3.21±0.09
	Ethylene Glycol	36.41±9.18	38.41±13.83	33.82±2.60	30.02±2.88	34.21±3.52	29.00±2.19	7.40±0.73	7.83±2.66	5.55±0.41	5.64±0.39	5.37±0.45	6.36±0.25
	Fructofuranose	12.37±1.66	5.92±0.80	152.39±56.80	36.88±5.38	338.93±23.19	346.96±24.28	3.88±1.07	2.51±1.09	2.28±0.44	4.00±0.87	6.42±1.40	3.54±0.46
	Fructose	13.35±4.02	0.80±0.17	197.63±42.26	22.40±4.65	298.39±79.86	412.11±263.01	1.25±0.28	0.81±0.10	1.58±0.23	1.77±0.14	4.33±0.42	1.60±0.24
	Galactinol	12.50±1.10	17.64±1.54	41.73±5.07	59.61±13.56	78.02±20.74	57.99±10.02	2.18±0.19	2.04±1.11	1.79±0.27	3.34±0.53	1.41±0.33	1.69±0.45
	Glucose	22.44±6.33	10.36±1.94	134.01±40.10	46.68±9.63	285.81±24.32	231.95±101.60	3.42±0.61	2.86±0.75	2.95±0.24	4.72±2.86	6.15±1.30	4.09±0.32
	Glycerol	19.34±2.81	12.64±3.03	59.95±24.85	48.19±2.93	38.76±12.14	34.72±5.17	6.68±0.72	7.38±0.72	6.65±0.51	7.24±0.81	6.73±0.87	6.89±0.93
	Inositol, Myo-	10.48±1.52	10.83±1.77	46.05±9.41	45.00±5.16	50.85±4.83	48.80±6.28	2.33±0.66	2.08±0.10	2.70±0.51	4.80±1.52	3.66±0.49	5.35±0.73
	Inositol-2-Phosphate	43.35±1.86	38.52±5.06	74.93±2.91	68.30±19.19	78.65±12.44	79.23±7.74	17.23±5.47	17.34±1.41	13.99±0.63	12.75±4.01	14.41±2.74	20.12±1.33
	Lactose	32.46±3.92	30.51±1.62	81.91±12.78	86.28±17.35	77.66±9.01	71.62±6.33	8.72±0.39	7.70±0.25	9.12±1.73	9.13±0.91	7.40±0.57	8.77±0.32
	Lyxos	13.15±2.20	9.03±0.20	85.90±2.90	82.71±25.99	82.76±25.71	54.08±9.19	3.47±1.11	4.04±0.49	2.91±0.73	3.62±0.94	4.59±0.30	5.19±0.35
Sugars	Maltose	31.12±6.71	33.94±2.07	135.92±49.38	180.28±37.05	155.16±34.06	108.14±9.91	6.75±3.60	4.15±0.09	3.62±0.33	6.07±0.53	3.27±0.33	4.01±0.06
	Mannitol	14.04±2.67	8.69±0.80	39.44±1.36	37.39±11.68	39.14±3.99	38.39±4.66	11.29±9.14	5.07±1.92	38.89±6.44	15.62±2.19	14.60±2.81	15.38±2.20
	Mannose	10.40±2.02	6.36±0.74	63.25±17.52	33.71±4.68	80.20±5.83	101.42±6.00	3.48±0.37	2.93±0.52	2.96±0.30	3.56±1.48	2.85±0.19	4.94±0.89
	Melezitose	9.70±0.65	7.83±0.90	158.88±8.04	111.52±9.90	75.10±14.95	74.30±9.89	4.08±1.15	2.53±0.15	2.95±0.51	3.67±0.78	3.44±1.22	4.75±2.14
	Pentitol	2.16±0.31	4.40±0.44	7.29±0.12	50.63±5.71	47.59±8.91	20.61±4.46	0.22±0.05	0.31±0.03	0.71±0.16	1.20±0.26	1.63±0.40	2.50±0.87
	Raffinose	6.41±1.32	6.27±1.07	110.14±26.46	123.10±19.76	97.53±15.75	76.22±8.63	0.15±0.04	0.07±0.02	0.48±0.08	0.76±0.17	0.21±0.04	0.41±0.06
	Salicylic Acid-Glucopyranoside	10.54±6.07	11.53±2.63	37.16±17.42	29.01±4.55	47.14±16.02	23.71±1.33	0.11±0.05	0.09±0.01	0.07±0.06	0.08±0.02	0.07±0.04	0.09±0.08
	Sorbitol	12.02±2.51	12.66±0.99	132.12±51.40	109.19±14.15	120.84±32.34	83.22±8.94	4.80±1.33	4.64±0.51	5.54±0.16	8.17±4.33	5.76±1.17	5.02±0.62
	Sucrose	22.20±1.11	16.73±1.82	49.45±8.42	44.41±5.54	44.70±6.19	44.88±5.05	5.94±0.24	6.08±0.05	7.29±0.12	7.52±0.95	5.23±0.73	6.06±0.81
	Trehalose	3.07±1.68	1.70±0.05	4.22±0.44	4.96±1.29	6.27±0.91	6.85±1.04	14.79±1.88	13.85±1.74	35.70±1.74	30.22±7.75	60.24±12.69	81.84±7.59
	Xylitol	45.25±14.93	31.34±15.80	101.51±22.42	61.95±11.23	58.68±8.52	45.59±4.82	2.79±0.10	5.31±0.89	0.33±0.03	1.38±0.42	0.70±0.24	4.64±1.46
	Xylose	10.29±3.58	5.06±0.93	99.04±21.85	95.60±9.13	92.85±22.41	67.74±24.57	5.82±1.89	5.25±0.21	2.49±0.93	3.10±1.33	2.44±0.40	2.37±0.31

	4-Hydroxy-Cinnamic Acid	24.37±2.75	24.26±0.79	45.33±3.69	56.35±9.15	50.41±4.36	47.64±11.60	8.02±1.34	8.13±0.18	9.07±1.63	9.69±1.72	13.10±1.46	13.24±0.96
	3-Deoxy-Arabino-Hexaric Acid	23.89±2.81	21.94±2.51	40.04±2.67	40.70±2.40	40.22±6.95	43.39±4.49	12.98±0.28	15.02±0.63	8.29±1.42	9.85±2.89	9.94±2.32	12.44±0.92
	Acetic Acid	27.72±2.72	48.73±2.11	59.61±2.02	58.81±9.85	54.26±3.36	55.37±10.41	15.27±2.43	17.82±3.28	8.63±0.97	7.05±0.98	12.75±1.26	12.83±1.76
	Benzoic Acid	40.15±4.97	64.71±15.84	40.91±4.17	73.86±13.48	32.56±1.38	35.29±5.08	10.52±1.62	11.30±2.59	7.73±1.40	9.85±0.72	9.10±1.67	10.48±0.74
	Beta-D-Glucopyranuronic Acid	22.00±4.57	17.92±2.50	33.44±4.93	32.82±8.19	46.65±4.74	33.00±5.04	6.79±1.51	7.90±0.25	4.91±0.55	5.89±1.35	3.06±0.69	3.41±0.57
	Citric Acid	31.40±1.26	32.15±1.63	31.03±11.06	28.09±5.59	44.94±4.30	47.21±3.39	0.50±0.13	0.56±0.02	0.53±0.01	0.39±0.10	0.49±0.04	0.83±0.08
	Eicosanoic Acid	18.96±1.28	28.57±1.36	24.55±0.13	44.59±5.56	47.58±10.77	24.67±1.11	14.38±2.04	13.59±3.34	16.35±1.94	25.54±3.08	15.82±1.22	13.77±1.26
	Erythronic Acid	19.76±2.24	20.50±0.48	75.96±17.45	59.66±10.25	74.00±9.70	57.04±16.32	5.10±0.71	4.78±0.58	4.21±0.64	2.43±0.48	3.31±0.43	3.66±0.09
	Fumaric Acid	34.05±2.06	35.17±2.29	45.32±4.23	50.87±5.23	47.41±3.21	42.81±4.25	8.02±3.58	9.10±0.20	3.81±0.71	5.29±0.34	4.03±0.28	6.14±0.94
	Gamma.-Hydroxybutyric Acid	18.56±1.17	84.64±5.79	23.32±5.77	102.40±8.31	37.65±1.57	25.89±12.80	11.63±1.42	14.14±0.31	12.01±0.97	15.13±1.32	12.75±1.63	11.48±0.87
Organic acids	Gluconic Acid	42.75±5.27	48.46±3.30	88.99±10.82	136.13±8.41	96.23±11.87	95.18±14.77	8.33±1.27	5.28±0.23	5.73±1.34	5.30±1.48	8.84±1.34	11.23±3.49
	Glucopyranose	17.26±4.47	7.12±0.97	87.08±26.57	29.94±5.56	155.77±33.18	139.04±52.15	2.88±0.65	2.37±0.70	2.51±0.21	3.68±1.23	4.93±0.82	3.44±0.26
	Glutamic Acid	104.41±12.91	47.98±5.06	116.47±19.59	26.47±3.69	44.43±4.20	121.21±9.08	25.15±9.35	8.13±0.24	25.58±3.78	7.27±3.62	42.36±4.10	49.64±10.22
	Glyceric Acid	21.60±3.59	16.83±0.35	88.27±23.26	79.93±5.66	70.14±4.04	67.06±16.10	2.10±0.46	2.48±1.06	2.51±0.16	2.28±0.20	1.75±0.32	2.23±0.06
	Hexadecanoic Acid	17.75±2.63	27.11±1.95	16.73±0.24	28.42±2.11	23.80±2.36	18.26±4.95	9.41±0.77	8.70±0.72	11.88±1.39	15.73±0.84	10.87±0.83	9.41±0.43
	Isocitric Acid	22.59±3.59	27.02±1.71	26.81±8.48	36.95±1.51	50.39±6.24	49.73±5.46	0.47±0.08	0.40±0.07	0.45±0.01	0.26±0.12	0.44±0.04	0.73±0.07
	Malic Acid	28.47±0.53	24.13±2.16	24.38±3.42	22.98±2.19	24.82±1.04	19.35±3.52	9.16±3.51	10.56±0.99	3.15±0.52	3.80±0.90	3.74±0.69	4.87±0.64
	Octadecanoic Acid	17.76±2.41	35.09±2.51	15.80±1.27	34.80±2.51	26.43±3.16	18.36±5.55	11.58±0.80	11.59±0.29	14.41±1.53	23.83±3.37	13.00±1.18	11.13±0.48
	Octadecatrienoic Acid	44.07±3.68	39.29±5.75	39.32±2.20	32.25±7.15	39.41±7.47	31.79±12.78	4.93±0.89	5.32±0.16	4.23±0.65	5.15±0.83	4.07±0.49	5.07±0.39
	Oxalic Acid	49.99±3.36	46.30±9.71	17.57±7.76	18.01±6.29	33.52±0.36	49.15±2.39	0.30±0.06	0.29±0.03	0.31±0.05	0.53±0.06	0.35±0.05	1.09±0.15
	Phosphoric Acid	36.15±5.61	38.56±3.47	32.46±2.21	28.80±2.30	36.35±1.39	34.30±5.76	14.35±1.73	14.50±0.89	14.73±2.98	11.95±1.56	13.64±2.54	15.57±1.71
	Pipecolic Acid	17.73±1.77	25.13±0.77	42.67±1.10	54.46±7.46	45.84±3.42	36.18±3.15	3.67±0.28	3.94±0.09	3.29±0.59	3.58±0.43	4.41±0.59	5.85±1.76
	Propanoic Acid	28.05±1.55	45.62±7.61	37.35±4.91	38.74±3.64	19.68±2.29	28.11±6.16	41.54±5.94	17.41±9.48	13.35±1.14	10.37±7.35	16.20±2.06	12.37±5.11

	Pyruvic Acid	42.96±4.35	24.34±2.74	45.86±7.13	17.81±2.22	30.26±4.86	55.09±5.17	9.85±0.85	7.87±0.64	7.64±0.83	6.63±0.59	6.92±0.91	7.81±0.35
	Quinic Acid	14.60±2.79	12.05±0.66	36.93±0.06	48.45±9.52	46.60±11.50	54.26±6.54	5.72±0.63	5.13±0.64	3.59±1.03	5.64±2.25	2.58±0.33	6.36±0.31
	Saccharic Acid	40.79±7.62	50.30±7.65	97.19±1.74	127.88±24.42	116.55±6.31	116.05±21.31	3.77±0.27	4.46±0.06	4.11±0.54	2.81±1.03	3.07±0.67	3.63±0.76
	Salicylic Acid	69.36±7.86	40.65±1.33	50.65±5.81	50.14±14.75	50.26±7.83	47.38±7.16	0.25±0.06	0.34±0.06	0.15±0.03	0.29±0.04	0.25±0.05	0.33±0.11
	Succinic Acid	36.85±2.51	33.08±1.94	55.59±17.31	53.25±6.23	36.36±1.07	34.44±8.80	5.39±2.25	4.96±0.41	2.42±0.58	2.36±0.28	3.13±0.16	2.93±0.64
	Threonic Acid	13.56±0.82	6.66±0.48	125.81±74.05	100.94±14.26	81.69±13.04	66.36±24.03	1.20±0.70	0.33±0.00	0.73±0.01	0.85±0.13	0.67±0.09	0.87±0.19
Others	1-Monohexadecanoylglycerol	15.94±3.01	20.87±0.03	19.61±2.41	26.97±2.47	23.16±3.61	17.64±4.01	8.71±0.76	6.65±0.98	9.71±1.06	11.53±1.70	9.12±1.02	8.40±0.19
	1-Monooctadecanoylglycerol	18.47±2.56	25.71±1.53	22.72±1.98	36.04±2.94	28.13±3.64	22.46±3.83	10.22±0.90	8.21±0.76	12.22±1.77	14.31±2.22	11.22±0.94	10.47±0.36
	2-Aminoethanol	21.49±2.54	13.32±1.49	55.94±14.51	56.37±1.48	45.94±6.98	41.14±10.40	10.47±2.08	13.65±0.97	12.13±1.70	14.96±1.18	36.76±5.86	44.72±9.76
	5-Hydroxy-Tryptamine,	2.32±0.16	15.30±3.36	6.71±3.03	45.98±16.45	17.26±2.76	6.10±0.57	35.04±17.30	105.44±0.42	38.02±13.41	79.28±25.52	80.57±13.14	92.85±4.24
	Adenosine	7.48±2.28	1.88±0.85	138.54±32.34	84.48±37.50	139.71±41.91	76.67±17.47	1.53±0.17	1.17±0.10	0.68±0.06	1.52±0.26	1.04±0.24	0.91±0.17
	Allantoin	7.21±7.88	6.68±9.43	14.38±0.12	34.98±14.49	81.22±2.54	34.65±10.36	7.83±6.30	21.36±1.92	872.9±214.1	20.48±3.94	451.8±58.6	894.3±264.2
	Campesterol	59.02±13.39	60.57±6.45	32.82±15.15	39.68±11.87	62.91±0.04	51.42±41.41	26.44±4.56	27.10±6.68	24.99±5.10	29.28±4.29	24.87±5.72	28.80±1.31
	Erythritol	33.85±7.34	31.27±1.90	81.52±20.13	47.60±6.96	68.94±3.40	55.46±28.11	4.02±0.22	4.09±0.86	2.59±0.90	2.84±0.57	4.21±1.40	2.72±0.13
	Ethanolanine	38.75±9.86	27.60±2.68	21.25±0.56	61.62±12.16	59.85±8.25	19.18±6.05	12.21±2.56	17.39±2.89	14.29±1.38	25.42±2.69	49.05±10.05	59.48±11.00
	Formylpiperidine	41.58±7.29	40.21±2.12	39.74±1.49	41.41±0.93	24.42±2.58	33.72±4.04	10.76±3.19	13.33±1.52	10.03±0.89	12.17±1.35	10.01±1.33	12.17±0.46
	Galactosylglycerol	17.03±6.23	5.63±0.43	128.89±73.62	67.27±26.55	78.62±3.41	49.23±13.80	2.12±1.08	1.77±0.25	2.54±0.91	3.18±0.50	2.05±0.06	2.64±0.54
	Threonic Acid-1,4-Lactone	19.00±1.36	12.91±0.97	135.00±83.41	76.85±6.35	55.79±20.89	71.61±9.83	2.33±0.45	1.04±0.27	1.22±0.06	0.99±0.38	1.57±0.27	2.20±0.56
	Triethanolamine	10.26±1.99	6.73±0.36	12.10±4.67	7.15±0.28	11.37±0.28	13.51±7.28	4.67±1.02	4.23±0.25	5.63±1.56	4.40±0.36	4.20±0.98	3.89±0.41
	Uracil	30.03±5.02	29.14±1.99	58.32±21.64	67.59±6.87	38.97±1.19	38.39±5.04	10.42±1.68	11.68±0.07	8.47±0.92	12.90±0.95	7.82±1.22	10.27±1.53
	Urea	20.33±4.19	10.79±0.55	28.54±7.01	26.13±2.81	33.30±4.65	25.44±4.66	17.57±6.64	0.15±0.07	12.01±1.94	7.34±0.78	12.77±1.02	4.55±0.47
	Vitamin E	110.60±25.35	106.81±14.38	4.45±0.32	20.50±7.05	51.64±28.01	9.55±1.71	1.31±0.30	0.89±0.19	1.31±0.35	1.78±0.27	1.59±0.19	1.23±0.03

Table S3. Morphological and yield traits performances of the IR64 and PL177 measured under control, salt and salt plus ABA conditions

Genotype	Treatment	Plant height (cm)	Tiller number	Grain weight per plant (g/plant)	SES	Shoot length (cm)	Root length (cm)	Shoot dry weight (mg/plant)	Root dry weight (mg/plant)	Relative water content (%)
IR64	Control	99.8±1.5 [a, p]	12.1±0.5 [a, p]	16.9±1.1 [a, p]	1.0±0[a, p]	32.8±0.1[a, p]	10.1±0.7[a, p]	46.5±1.7[a, p]	6.84±0.94[a, p]	83.7±5.1[a, p]
	Salt	65.3±1.3 [b, p]	5.6±0.6 [b, p]	5.1±0.7 [b, p]	5.0±0.5[c, p]	18.0±0.8[b, p]	9.0±0.7[a, p]	20.1±1.3[c, p]	4.83±0.20[b, p]	64.5±3.7[b, p]
	Salt+ABA	_	_	_	3.0±0.0[b, p]	19.1±0.9[b, p]	9.2±0.3[a, p]	33.4±0.7[b, p]	6.72±0.63[a, p]	86.6±1.0[a, p]
PL177	Control	103.4±2.8 [a, p]	11.5±0.6 [a, p]	17±0.9 [a, p]	1.0±0[a, p]	34.5±0.9[a,p]	10.0±0.3[a, p]	46.6±2.8[a, p]	7.23±0.79[a, p]	82±3.0[a, p]
	Salt	71.8±1.6 [b, q]	7.5±0.6 [b, q]	7.9±0.7 [b, q]	3.8±0.3[c, q]	24.8±1.0[b, q]	9.5±0.2[a, p]	27.2±2.6[c, q]	6.41±0.49[a, q]	76.9±2.1[b, q]
	Salt+ABA	_	_	_	3.0±0.0[b, p]	19.3±0.8[c, p]	9.4±0.5[a, p]	35.2±2.2[b, p]	6.48±0.60[a, p]	87.7±3.7[a, p]

Different letters (a, b, c) within different conditions of ONE genotype (IR64 OR PL177) are significantly different (Fisher LSD, $p \leqslant 0.05$), whereas different letters (p, q) between two genotypes under ONE condition (control OR salinity OR salinity+ABA) are significantly different(Fisher LSD, $p \leqslant 0.05$)

Table S4. Shoots and roots sodium and potassium concentrations in IR64 and PL177 under salt and salt+ABA treatment conditions

Genotype	Treatment	Shoot K^+ concentration (mmol/g dry weight)	Shoot Na^+ concentration (mmol/g dry weight)	Na^+/K^+ ratio in shoot	Root K^+ concentration (mmol/g dry weight)	Root Na^+ concentration (mmol/g dry weight)	Na^+/K^+ ratio in roots	Na^+ concentration in roots to shoot translocation
IR64	Control	1.02±0.04$^{a, p}$	0.03±0.01$^{a, p}$	0.03$^{a, p}$	0.66±0.08$^{a, p}$	0.06±0.01$^{a, p}$	0.09$^{a, p}$	0.45$^{a, p}$
PL177	Control	1.02±0.03$^{a, p}$	0.04±0.01$^{a, p}$	0.03$^{a, p}$	0.72±0.03$^{a, p}$	0.07±0.01$^{a, p}$	0.09$^{a, p}$	0.53$^{a, p}$
IR64	Salt	0.81±0.04$^{b, p}$	1.31±0.11$^{c, p}$	1.63$^{a, q}$	0.23±0.02$^{b, p}$	0.63±0.05$^{b, p}$	2.71$^{a, q}$	2.09$^{a, q}$
PL177	Salt	0.77±0.05$^{c, p}$	0.96±0.05$^{c, q}$	1.25$^{a, p}$	0.22±0.02$^{b, p}$	0.67±0.05$^{b, p}$	3.01$^{a, p}$	1.45$^{a, p}$
IR64	Salt+ABA	0.95±0.03$^{a, p}$	0.84±0.09$^{b, p}$	0.89$^{a, p}$	0.22±0.02$^{b, p}$	0.87±0.14$^{c, p}$	3.94$^{a, p}$	1.00$^{a, p}$
PL177	Salt+ABA	0.89±0.08$^{b, p}$	0.90±0.01$^{b, p}$	1.01$^{a, q}$	0.23±0.04$^{b, p}$	0.88±0.09$^{c, p}$	3.82$^{a, p}$	1.02$^{a, p}$

Different letters of a,b, c represent significant difference (LSD, $p \leqslant 0.05$) between the three treatments of the same genotype (IR64 or PL177),whereas different letters of p and q represent significantly difference t(LSD, $p \leqslant 0.05$) between the two genotypes under the same treatment (control, salinity or salinity+ABA), respectively.

Table S5 Summary of the fold changes of 88 primary metabolites in two genotypes (PL177 and IR64) in response to the salt stress and salt+ABA treatments at the seedling stage

		n	Salt/Control				Salt+ABA/Control			
			PL177		IR64		PL177		IR64	
			Mean±SD	Range	Mean±SD	Range	Mean±SD	Range	Mean±SD	Range
Shoots	AAs	19	6.70±14.44	1.01～66.01	15.07±45.79	1.24～203.8	5.05±5.94	-1.16～25.31	44.6±175.4	-1.00～768.9
	OAs	18	1.22±2.30	-2.84～9.28	1.41±3.27	-2.57～15.16	1.16±2.03	-2.35～6.03	1.13±2.59	-3.27～9.96
	Sugars	25	5.47±5.26	-3.10～17.18	6.41±7.27	-5.75～28.11	7.32±7.51	-1.49～27.40	28.5±102.1	-2.18～515.1
	Others	16	1.28±8.50	-24.9～18.5	5.12±11.22	-5.21～44.94	3.43±5.17	-2.14～18.65	3.19±11.0	-11.18～40.8
	Total	88	3.62±8.41	-24.9～66.0	6.45±22.32	-5.75～203.8	4.16±5.90	-2.35～27.40	18.7±97.9	-11.2～768.9
Roots	AAs	19	1.45±2.05	-1.32～7.81	1.52±2.04	-1.34～8.37	2.00±2.72	-1.40～10.34	4.03±4.44	-1.27～15.87
	OAs	18	-0.59±1.47	-3.11～1.26	-0.53±1.58	-2.78～2.54	-0.65±1.40	-2.56～1.68	0.13±2.02	-2.31～6.11
	Sugars	25	-0.41±2.47	-8.53～3.44	0.89±2.74	-3.86～10.07	0.37±2.47	-3.99～7.41	1.15±2.54	-2.22～8.20
	Others	16	6.91±28.03	-2.26～111.9	3.58±12.16	-1.44～48.95	3.88±14.47	-1.49～57.7	5.01±12.4	-1.51～41.8
	Total	88	1.27±12.10	-8.53～111.9	1.06±5.59	-3.86～48.95	1.04±6.53	-3.99～57.7	2.15±6.13	-2.31～41.8

Table S6-A. ANOVA results for 88 primary metabolites in shoots of two rice genotypes (G), IR64 and PL177, measured under three different treatments (Trt), control, salt and salt+ABA(SA)

	Genotype (G)				Treatment (T)				G x T			
	MS	F Value	Pr > F	R^2(%)	MS	F Value	Pr > F	R^2(%)	MS	F Value	Pr > F	R^2(%)
Formylpiperidine	48.1	2.9	0.1132	**5.0**	299.6	17.8	0.0001	**62.4**	51.4	3.1	0.0795	**10.7**
2-Aminoethanol	82.2	1.7	0.2088	**1.4**	2437.6	51.5	0.0001	**84.0**	27.8	0.6	0.5692	**1.0**
Inositol-2-Phosphate	61.8	0.5	0.4828	**0.9**	2738.2	23.0	0.0001	**76.2**	22.2	0.2	0.8315	**0.6**
4-Amino- Butyric Acid	784.7	9.6	0.0079	**6.6**	5214.7	63.6	0.0001	**87.7**	60.9	0.7	0.4938	**1.0**
5-Hydroxy-Tryptamine,	880.8	14.4	0.0020	**15.3**	511.8	8.3	0.0041	**17.7**	955.9	15.6	0.0003	**33.1**
Gamma.-Hydroxybutyric Acid	9283.6	160.8	0.0001	**39.4**	1534.8	26.6	0.0001	**13.0**	3890.7	67.4	0.0001	**33.0**
Galactosylglycerol	5472.0	9.3	0.0087	**15.9**	11759.8	20.0	0.0001	**68.2**	954.3	1.6	0.2329	**5.5**
Cytosine	244.7	7.2	0.0182	**7.7**	1301.8	38.0	0.0001	**81.5**	30.7	0.9	0.4298	**1.9**
Phenylalanine	640.0	1.0	0.3378	**2.2**	9449.9	14.6	0.0004	**65.9**	606.1	0.9	0.4165	**4.2**
Benzoic Acid	1894.5	21.7	0.0004	**29.4**	974.6	11.2	0.0013	**30.3**	383.2	4.4	0.0329	**11.9**
4-Hydroxy-Cinnamic Acid	34.6	0.7	0.4293	**0.8**	1432.2	27.5	0.0001	**69.9**	77.6	1.5	0.2597	**3.8**
Beta-D-Glucopyranuronic Acid	175.8	5.8	0.0299	**8.7**	696.4	23.1	0.0001	**68.6**	71.9	2.4	0.1282	**7.1**
Glucopyranose	3682.2	4.6	0.0501	**4.5**	32212.2	40.2	0.0001	**78.2**	935.8	1.2	0.3396	**2.3**
Alanine	900.7	18.0	0.0008	**13.0**	2806.3	56.1	0.0001	**80.9**	299.9	6.0	0.0132	**8.6**
Propanoic Acid	391.2	17.6	0.0009	**23.3**	400.4	18.0	0.0001	**47.7**	100.4	4.5	0.0306	**12.0**
Pyruvic Acid	249.2	13.2	0.0027	**6.3**	216.5	11.4	0.0011	**11.0**	1275.2	67.4	0.0001	**64.7**
Campesterol	5.0	0.0	0.9186	**0.1**	960.6	2.1	0.1599	**22.5**	140.0	0.3	0.7416	**3.3**
Oxalic Acid	80.0	2.7	0.1240	**1.9**	1473.6	49.3	0.0001	**70.6**	174.5	5.8	0.0143	**8.4**
Erythritol	1303.4	5.9	0.0297	**15.5**	2072.6	9.3	0.0027	**49.3**	372.8	1.7	0.2225	**8.9**
Erythronic Acid	551.7	4.8	0.0467	**4.8**	4729.9	40.8	0.0001	**82.1**	166.3	1.4	0.2710	**2.9**
1-Monooctadecanoylglycerol	115.5	13.0	0.0029	**13.8**	79.8	9.0	0.0031	**19.0**	146.7	16.5	0.0002	**35.0**
Eicosanoic Acid	23.8	1.0	0.3375	**0.9**	303.0	12.6	0.0007	**22.1**	795.1	33.0	0.0001	**58.1**

Fructofuranose	6772.4	15.4	0.0015	**1.5**	203734.8	462.5	0.0001	**89.1**	6583.4	15.0	0.0003	**2.9**
D-Xylofuranose	176.6	3.2	0.0933	**11.9**	73.1	1.3	0.2930	**9.8**	126.9	2.3	0.1337	**17.1**
Proline	47491.3	18.7	0.0007	**4.8**	290237.5	114.0	0.0001	**58.2**	209566.3	82.3	0.0001	**42.0**
Glycine	244.8	4.9	0.0436	**3.5**	3140.7	63.1	0.0001	**89.9**	26.2	0.5	0.6020	**0.7**
Mannose	79.7	2.0	0.1806	**0.3**	11651.6	290.2	0.0001	**85.6**	971.5	24.2	0.0001	**7.1**
Mannitol	34.6	0.9	0.3554	**0.9**	1629.3	43.0	0.0001	**85.2**	9.5	0.3	0.7814	**0.5**
Glycerol	264.2	3.5	0.0829	**4.8**	2211.7	29.2	0.0001	**80.3**	22.7	0.3	0.7458	**0.8**
Glyceric Acid	136.7	1.3	0.2757	**0.8**	7283.3	68.6	0.0001	**89.5**	10.6	0.1	0.9056	**0.1**
Glutamic Acid	2530.5	28.1	0.0001	**8.1**	199.4	2.2	0.1456	**1.3**	12421.8	138.1	0.0001	**79.9**
Glutamine	84310.6	19.1	0.0006	**11.0**	129944.2	29.4	0.0001	**34.0**	94744.1	21.4	0.0001	**24.8**
Trehalose	0.0	0.0	0.9779	**0.0**	29.9	22.5	0.0001	**72.4**	2.2	1.7	0.2195	**5.4**
Succinic Acid	33.8	0.7	0.4173	**1.5**	710.8	14.7	0.0004	**64.7**	1.6	0.0	0.9678	**0.1**
Inositol, Myo-	4.0	0.2	0.6952	**0.1**	3078.7	124.3	0.0001	**92.4**	2.5	0.1	0.9060	**0.1**
Methionine	17.4	1.5	0.2468	**5.6**	17.0	1.4	0.2717	**11.0**	33.6	2.8	0.0927	**21.8**
Arginine	9.7	0.1	0.7414	**0.1**	5762.0	67.4	0.0001	**71.6**	1000.1	11.7	0.0010	**12.4**
Quinic Acid	144.3	2.9	0.1089	**2.2**	2584.4	52.5	0.0001	**79.1**	83.2	1.7	0.2200	**2.5**
Lyxos	675.5	2.6	0.1285	**2.8**	9445.6	36.5	0.0001	**78.3**	342.3	1.3	0.2978	**2.8**
Tyrosine	8.8	0.2	0.6462	**0.1**	3808.3	95.6	0.0001	**88.3**	62.1	1.6	0.2449	**1.4**
Tyramine	92.3	0.7	0.4128	**2.9**	556.0	4.3	0.0351	**35.1**	110.5	0.9	0.4469	**7.0**
Leucine	14215.5	2.6	0.1263	**8.9**	30632.7	5.7	0.0155	**38.3**	7529.4	1.4	0.2792	**9.4**
Phosphoric Acid	5.7	0.3	0.5763	**1.3**	69.6	4.0	0.0419	**30.9**	15.6	0.9	0.4295	**6.9**
Maltose	0.0	0.0	0.9968	**0.0**	27935.4	42.0	0.0001	**71.2**	3184.5	4.8	0.0261	**8.1**
Raffinose	37.6	0.2	0.6596	**0.1**	20532.5	110.6	0.0001	**85.9**	461.4	2.5	0.1192	**1.9**
Xylose	594.9	2.3	0.1496	**1.7**	14502.7	56.7	0.0001	**85.0**	235.4	0.9	0.4214	**1.4**
Xylitol	2311.3	14.3	0.0020	**25.0**	2889.0	17.9	0.0001	**62.6**	326.4	2.0	0.1698	**7.1**
Uracil	31.7	0.6	0.4613	**0.6**	1736.2	31.5	0.0001	**68.9**	48.1	0.9	0.4398	**1.9**
Citric Acid	0.0	0.0	0.9897	**0.0**	519.1	24.4	0.0001	**70.7**	10.5	0.5	0.6205	**1.4**

Pipecolic Acid	47.4	2.9	0.1127	**1.3**	1234.7	74.7	0.0001	**67.5**	204.9	12.4	0.0008	**11.2**
Malic Acid	65.6	12.2	0.0036	**26.4**	30.9	5.7	0.0153	**24.9**	6.4	1.2	0.3322	**5.2**
Gluconic Acid	1399.5	14.3	0.0020	**5.9**	7673.6	78.4	0.0001	**64.5**	983.6	10.1	0.0020	**8.3**
Lactose	6.9	0.1	0.7972	**0.1**	5042.7	50.4	0.0001	**83.2**	40.7	0.4	0.6735	**0.7**
Triethanolamine	21.0	1.5	0.2373	**6.5**	28.0	2.0	0.1678	**17.4**	22.4	1.6	0.2317	**14.0**
Tryptophan	10790.6	5.3	0.0369	**3.0**	117490.9	57.9	0.0001	**64.6**	44946.5	22.2	0.0001	**24.7**
Sorbitol	1872.5	4.7	0.0482	**3.7**	21523.5	53.9	0.0001	**84.6**	637.0	1.6	0.2378	**2.5**
Octadecanoic Acid	416.6	35.8	0.0001	**29.5**	29.3	2.5	0.1162	**4.2**	375.5	32.2	0.0001	**53.1**
Hexadecanoic Acid	125.6	13.9	0.0022	**22.3**	4.7	0.5	0.6023	**1.7**	141.2	15.7	0.0003	**50.2**
1-Monohexadecanoylglycerol	23.9	2.7	0.1249	**5.5**	35.8	4.0	0.0427	**16.6**	75.0	8.4	0.0041	**34.7**
Salicylic Acid	537.7	6.5	0.0228	**18.8**	70.1	0.9	0.4475	**4.9**	398.2	4.8	0.0252	**27.9**
Serine	24.6	0.1	0.7341	**0.1**	16729.0	81.8	0.0001	**89.8**	30.9	0.2	0.8612	**0.2**
Melezitose	1306.2	16.6	0.0011	**2.6**	24226.1	307.8	0.0001	**97.0**	1017.2	12.9	0.0007	**4.1**
Threonine	534.5	4.7	0.0472	**2.2**	11068.1	98.0	0.0001	**92.6**	26.2	0.2	0.7956	**0.2**
Threonic Acid	1157.5	2.0	0.1808	**2.7**	16766.3	28.7	0.0001	**79.5**	121.4	0.2	0.8146	**0.6**
Threonic Acid-1,4-Lactone	1223.6	2.1	0.1715	**3.6**	12297.0	20.9	0.0001	**71.4**	2111.6	3.6	0.0553	**12.3**
Saccharic Acid	822.3	3.3	0.0923	**2.9**	10500.0	41.7	0.0001	**73.5**	371.9	1.5	0.2619	**2.6**
Aspartic Acid	314.1	6.8	0.0210	**3.0**	4148.5	89.2	0.0001	**78.4**	243.7	5.2	0.0200	**4.6**
Asparagine	5152.7	58.7	0.0001	**10.9**	5680.7	64.7	0.0001	**23.9**	14549.1	165.7	0.0001	**61.3**
3-Deoxy-Arabino-Hexaric Acid	1.9	0.1	0.7348	**0.1**	734.8	47.3	0.0001	**83.2**	11.2	0.7	0.5027	**1.3**
Vitamin E	464.2	1.6	0.2270	**1.1**	16735.9	57.6	0.0001	**78.7**	1358.7	4.7	0.0279	**6.4**
Pentitol	180.5	2.4	0.1466	**2.0**	1830.8	24.0	0.0001	**39.7**	1855.9	24.3	0.0001	**40.2**
Adenosine	7856.1	11.3	0.0046	**12.2**	24188.5	34.9	0.0001	**75.3**	1601.1	2.3	0.1360	**5.0**
Valine	423.0	2.7	0.1219	**1.0**	19097.7	122.4	0.0001	**92.2**	95.7	0.6	0.5553	**0.5**
Octadecatrienoic Acid	197.7	3.2	0.0955	**15.1**	78.9	1.3	0.3100	**12.1**	3.8	0.1	0.9401	**0.6**
Fumaric Acid	2.2	0.0	0.8473	**0.1**	319.2	5.5	0.0172	**39.2**	39.5	0.7	0.5217	**4.9**
Ethanolanine	68.5	1.0	0.3432	**1.0**	118.8	1.7	0.2237	**3.4**	2482.5	34.9	0.0001	**71.5**

Ethylene Glycol	25.7	0.5	0.4872	**2.7**	70.6	1.4	0.2794	**15.0**	24.6	0.5	0.6248	**5.2**
Acetic Acid	237.0	4.9	0.0435	**7.1**	781.0	16.2	0.0002	**46.9**	237.1	4.9	0.0240	**14.2**
Isoleucine	1410.2	5.1	0.0403	**3.1**	20502.7	74.2	0.0001	**89.4**	253.5	0.9	0.4222	**1.1**
Sucrose	55.6	2.4	0.1450	**1.6**	1520.1	65.2	0.0001	**89.8**	16.4	0.7	0.5110	**1.0**
Fructose	2861.7	0.2	0.6775	**0.4**	217533.5	13.7	0.0005	**56.2**	31311.4	2.0	0.1758	**8.1**
Glucose	12256.4	5.0	0.0418	**4.7**	105085.9	43.1	0.0001	**80.0**	2166.6	0.9	0.4335	**1.6**
Isocitric Acid	100.8	4.9	0.0448	**3.6**	1152.2	55.5	0.0001	**82.4**	43.8	2.1	0.1582	**3.1**
Allantoin	366.3	3.9	0.0696	**2.7**	4573.3	48.2	0.0001	**67.3**	1844.0	19.4	0.0001	**27.2**
Urea	204.8	12.2	0.0035	**18.0**	372.5	22.3	0.0001	**65.4**	20.2	1.2	0.3290	**3.5**
Galactinol	4.7	0.0	0.8489	**0.0**	4963.1	39.8	0.0001	**76.2**	579.3	4.6	0.0284	**8.9**
1-Ethylglucopyranoside	1130.9	3.8	0.0729	**12.3**	1420.2	4.7	0.0270	**31.0**	27.6	0.1	0.9128	**0.6**
Salicylic Acid-Glucopyranoside	488.3	6.8	0.0208	**11.6**	1206.5	16.8	0.0002	**57.1**	260.1	3.6	0.0544	**12.3**

Table S6-B. ANOVA results for 88 primary metabolites in roots of two rice genotypes (G), IR64 and PL177, measured under three different treatments (Trt), control, salt and salt+ABA(SA)

	Genotype (G)				Treatment (T)				G x T			
	MS	F Value	Pr > F	R^2(%)	MS	F Value	Pr > F	R^2(%)	MS	F Value	Pr > F	R^2(%)
Formylpiperidine	24.6	7.6	0.0157	34.3	1.7	0.5	0.5960	4.9	0.1	0.0	0.9744	0.2
2-Aminoethanol	101.6	4.5	0.0526	2.6	1705.3	75.2	0.0001	87.5	13.6	0.6	0.5615	0.7
Inositol-2-Phosphate	11.0	0.9	0.3528	3.8	33.5	2.8	0.0940	23.1	22.9	1.9	0.1829	15.8
4-Amino- Butyric Acid	1.0	1.4	0.2651	6.1	1.6	2.2	0.1475	20.0	0.8	1.1	0.3492	10.3
5-Hydroxy-Tryptamine,	8014.1	29.8	0.0001	48.3	1361.1	5.1	0.0222	16.4	1274.1	4.7	0.0268	15.4
Gamma.-Hydroxybutyric Acid	9.9	6.1	0.0268	16.4	3.6	2.2	0.1444	12.0	9.4	5.8	0.0145	31.2
Galactosylglycerol	0.4	0.9	0.3664	3.8	1.3	2.7	0.0993	23.7	0.4	0.9	0.4126	8.2
Cytosine	0.0	0.0	0.9866	0.0	0.2	0.3	0.7421	3.8	0.5	0.7	0.5195	8.6
Phenylalanine	0.1	0.0	0.9072	0.0	443.6	51.3	0.0001	80.2	19.3	2.2	0.1442	3.5
Benzoic Acid	9.6	4.6	0.0509	18.4	6.7	3.2	0.0712	25.9	0.7	0.3	0.7255	2.6
4-Hydroxy-Cinnamic Acid	0.4	0.2	0.6629	0.3	44.4	22.2	0.0001	73.0	0.1	0.1	0.9355	0.2
Beta-D-Glucopyranuronic Acid	3.1	2.9	0.1101	4.5	25.6	23.8	0.0001	73.3	0.3	0.2	0.7893	0.7
Glucopyranose	0.4	0.6	0.4557	1.5	4.0	6.7	0.0093	33.9	3.1	5.2	0.0209	26.2
Alanine	3.3	9.0	0.0097	25.3	0.8	2.1	0.1618	11.7	1.6	4.3	0.0350	24.3
Propanoic Acid	499.4	2.3	0.1497	8.9	526.6	2.5	0.1223	18.8	206.3	1.0	0.4067	7.4
Pyruvic Acid	2.3	4.2	0.0604	6.9	5.0	9.1	0.0029	30.4	3.3	6.0	0.0129	20.1
Campesterol	41.1	1.8	0.1985	10.9	0.2	0.0	0.9895	0.1	5.7	0.3	0.7790	3.0
Oxalic Acid	0.5	81.3	0.0001	30.0	0.3	52.4	0.0001	38.7	0.2	39.8	0.0001	29.4
Erythritol	0.7	1.1	0.3175	3.8	2.8	4.2	0.0385	29.0	1.5	2.3	0.1368	16.1
Erythronic Acid	1.6	5.6	0.0333	7.3	4.6	16.3	0.0002	42.5	2.0	7.2	0.0072	18.7
1-Monooctadecanoylglycerol	0.2	0.1	0.7381	0.2	25.6	13.3	0.0006	55.9	6.9	3.6	0.0554	15.0

Eicosanoic Acid	21.1	4.4	0.0548	4.7	93.9	19.6	0.0001	41.9	63.3	13.2	0.0006	28.3
Fructofuranose	3.3	3.4	0.0859	6.5	7.2	7.4	0.0064	28.3	9.4	9.6	0.0024	36.7
D-Xylofuranose	3.3	18.4	0.0007	21.3	5.2	29.0	0.0001	66.9	0.1	0.7	0.5330	1.5
Proline	2.2	24.7	0.0002	27.4	2.2	24.0	0.0001	53.2	0.1	0.9	0.4433	1.9
Glycine	39.8	24.4	0.0002	34.2	13.6	8.3	0.0042	23.3	15.0	9.2	0.0028	25.8
Mannose	2.4	3.7	0.0763	12.9	1.0	1.5	0.2582	10.5	2.7	4.1	0.0398	28.8
Mannitol	430.2	1.3	0.2724	6.6	579.4	1.8	0.2084	17.8	259.9	0.8	0.4736	8.0
Glycerol	1.1	1.8	0.2041	10.9	0.1	0.1	0.8839	1.5	0.1	0.2	0.8100	2.6
Glyceric Acid	0.2	1.3	0.2722	5.9	0.3	1.8	0.1975	16.4	0.3	1.6	0.2386	14.3
Glutamic Acid	410.7	9.8	0.0075	7.7	1907.5	45.3	0.0001	71.2	344.0	8.2	0.0044	12.8
Glutamine	73.3	96.7	0.0001	12.7	165.4	218.1	0.0001	57.2	98.1	129.3	0.0001	33.9
Trehalose	120.3	2.1	0.1684	1.0	5211.9	91.4	0.0001	86.6	353.3	6.2	0.0118	5.9
Succinic Acid	0.3	0.2	0.6557	0.6	12.4	10.1	0.0019	53.9	0.1	0.0	0.9582	0.2
Inositol, Myo-	6.6	8.8	0.0104	16.8	8.1	10.8	0.0015	41.5	2.3	3.1	0.0784	11.8
Methionine	5.5	4.5	0.0523	17.5	1.1	0.9	0.4371	6.8	2.8	2.3	0.1372	17.9
Arginine	35.8	34.3	0.0001	9.7	137.4	131.6	0.0001	74.9	24.6	23.6	0.0001	13.4
Quinic Acid	14.4	10.3	0.0063	25.0	1.5	1.1	0.3610	5.3	7.2	5.1	0.0213	25.0
Lyxos	1.8	3.2	0.0972	9.7	4.7	8.1	0.0046	49.6	0.0	0.0	0.9844	0.1
Tyrosine	1.4	2.7	0.1228	6.1	5.9	11.8	0.0010	53.3	0.1	0.1	0.9007	0.5
Tyramine	0.7	0.2	0.6639	0.2	156.0	41.1	0.0001	78.5	20.3	5.4	0.0187	10.2
Leucine	0.9	3.3	0.0906	4.3	7.0	26.4	0.0001	69.2	0.9	3.6	0.0562	9.3
Phosphoric Acid	0.3	0.1	0.8085	0.3	3.2	0.7	0.4963	7.2	9.7	2.3	0.1416	22.1
Maltose	0.2	0.1	0.8035	0.2	5.3	1.8	0.1952	13.6	9.7	3.4	0.0646	24.7
Raffinose	0.1	2.9	0.1090	5.4	0.4	14.4	0.0004	53.5	0.1	1.8	0.1947	6.9
Xylose	0.0	0.0	0.9803	0.0	16.8	12.7	0.0007	57.8	0.5	0.4	0.6742	1.8
Xylitol	29.5	21.8	0.0004	37.0	15.7	11.6	0.0011	39.4	3.6	2.7	0.1056	9.0
Uracil	34.6	22.0	0.0003	39.6	7.3	4.6	0.0284	16.7	3.9	2.5	0.1179	9.0

Citric Acid	0.0	5.3	0.0369	8.1	0.1	9.7	0.0023	29.6	0.1	14.5	0.0004	44.3
Pipecolic Acid	2.1	3.3	0.0894	9.4	5.3	8.6	0.0037	48.4	0.7	1.2	0.3365	6.6
Malic Acid	5.3	1.7	0.2111	2.8	69.6	22.6	0.0001	73.5	0.2	0.1	0.9290	0.2
Gluconic Acid	0.6	0.2	0.6668	0.5	36.8	11.5	0.0011	54.5	11.2	3.5	0.0589	16.6
Lactose	0.1	0.1	0.7677	0.3	2.2	2.9	0.0857	22.1	2.2	3.0	0.0821	22.5
Triethanolamine	2.1	2.5	0.1382	11.9	1.6	1.9	0.1806	18.7	0.4	0.5	0.6214	4.8
Tryptophan	117.0	11.0	0.0051	19.9	132.1	12.4	0.0008	44.9	7.6	0.7	0.5070	2.6
Sorbitol	1.6	0.3	0.5749	1.6	7.4	1.6	0.2444	15.2	5.5	1.2	0.3455	11.2
Octadecanoic Acid	29.8	9.2	0.0091	6.1	116.2	35.7	0.0001	47.7	61.4	18.9	0.0001	25.2
Hexadecanoic Acid	1.5	1.9	0.1893	1.1	39.4	51.2	0.0001	60.9	13.8	17.9	0.0001	21.3
1-Monohexadecanoylglycerol	0.5	0.4	0.5346	0.9	13.6	11.4	0.0012	49.5	6.1	5.1	0.0223	22.0
Salicylic Acid	0.1	15.1	0.0017	44.7	0.0	3.1	0.0768	18.4	0.0	0.4	0.6531	2.6
Serine	0.2	0.4	0.5265	1.2	3.2	5.6	0.0163	32.8	2.6	4.5	0.0309	26.3
Melezitose	0.1	0.1	0.7681	0.5	1.4	0.9	0.4128	9.5	3.3	2.3	0.1367	23.1
Threonine	1.7	0.7	0.4341	1.5	30.7	11.7	0.0010	54.1	6.3	2.4	0.1271	11.1
Threonic Acid	0.1	1.3	0.2749	5.4	0.0	0.0	0.9915	0.1	0.5	4.4	0.0330	36.5
Threonic Acid-1,4-Lactone	0.4	3.0	0.1070	5.4	1.1	8.0	0.0049	28.8	1.4	9.9	0.0021	35.7
Saccharic Acid	0.0	0.0	0.9565	0.0	1.0	2.1	0.1593	15.8	2.0	4.3	0.0348	32.4
Aspartic Acid	3.7	2.2	0.1646	4.1	10.1	6.0	0.0135	22.7	21.1	12.4	0.0008	47.2
Asparagine	2.2	8.5	0.0112	5.8	12.2	47.0	0.0001	64.5	4.5	17.3	0.0002	23.8
3-Deoxy-Arabino-Hexaric Acid	19.4	5.7	0.0317	14.6	36.5	10.7	0.0015	54.8	0.4	0.1	0.8959	0.6
Vitamin E	0.1	0.9	0.3723	2.3	0.3	5.0	0.0236	27.4	0.4	6.4	0.0107	35.3
Pentitol	1.1	6.8	0.0206	7.7	5.1	32.0	0.0001	72.5	0.2	1.5	0.2673	3.3
Adenosine	0.1	1.6	0.2209	2.4	0.2	5.7	0.0154	16.9	0.6	17.4	0.0002	51.7
Valine	11.1	7.2	0.0182	8.2	43.9	28.2	0.0001	64.7	8.8	5.7	0.0158	13.0
Octadecatrienoic Acid	2.8	6.2	0.0256	26.2	0.5	1.1	0.3612	9.2	0.2	0.4	0.7076	3.0
Fumaric Acid	11.4	3.8	0.0711	10.5	27.4	9.2	0.0028	50.3	0.4	0.1	0.8727	0.8

Ethanolanine	373.0	8.7	0.0105	5.3	2980.8	69.7	0.0001	84.4	15.3	0.4	0.7050	0.4
Ethylene Glycol	1.2	1.6	0.2219	4.5	7.0	9.6	0.0024	52.3	0.4	0.5	0.6218	2.7
Acetic Acid	0.6	0.2	0.6738	0.2	116.4	36.9	0.0001	80.1	6.4	2.0	0.1672	4.4
Isoleucine	5.4	8.8	0.0103	3.8	60.6	98.7	0.0001	85.5	7.6	12.3	0.0008	10.7
Sucrose	0.7	1.8	0.2017	3.7	5.8	14.1	0.0005	58.2	0.2	0.6	0.5873	2.3
Fructose	4.6	61.2	0.0001	15.9	6.1	80.2	0.0001	41.6	4.0	52.9	0.0001	27.5
Glucose	0.4	0.2	0.6891	0.6	6.3	2.8	0.0963	21.4	6.4	2.8	0.0924	21.8
Isocitric Acid	0.0	0.1	0.7233	0.2	0.1	15.7	0.0003	40.4	0.1	17.0	0.0002	43.7
Allantoin	81978.3	4.8	0.0469	2.6	658768.3	38.2	0.0001	42.4	741563.2	43.0	0.0001	47.7
Urea	479.3	46.2	0.0001	65.8	2.0	0.2	0.8300	0.5	63.2	6.1	0.0125	17.3
Galactinol	1.5	6.7	0.0211	12.4	1.8	8.1	0.0046	29.7	1.2	5.6	0.0162	20.6
1-Ethylglucopyranoside	3.5	9.3	0.0086	11.6	8.4	22.3	0.0001	55.4	3.6	9.6	0.0024	23.8
Salicylic Acid-Glucopyranoside	0.0	0.0	0.9037	0.1	0.0	0.5	0.5977	6.8	0.0	0.2	0.8532	2.0

Table S7. The loadings (contributions) of each of the 88 metabolites to the first three principal components (PC)

		Shoots			Roots		
	Proportion of the total variance (%) explained	PC1 (60.4%)	PC2 (19.4%)	PC3 (11.3%)	PC1 (35.5%)	PC2 (26.3%)	PC3 (16.9%)
Amino acids	4-Amino- Butyric Acid	0.98	-0.12	-0.04	0.82	-0.12	0.49
	Cytosine	0.96	-0.09	-0.17	0.29	-0.47	0.42
	Phenylalanine	0.91	-0.13	-0.38	0.89	0.07	-0.17
	Alanine	0.88	-0.37	-0.21	0.53	0.55	0.59
	Proline	0.69	-0.56	-0.36	0.72	-0.27	-0.63
	Glycine	0.98	-0.15	-0.14	0.56	0.61	0.54
	Glutamine	0.55	0.75	-0.11	0.81	0.51	0.19
	Methionine	0.54	0.47	0.05	0.51	0.21	0.57
	Arginine	0.94	0.29	0.15	0.92	0.35	0.14
	Tyrosine	1.00	0.05	0.04	0.94	-0.15	0.19
	Tyramine	0.89	-0.20	0.00	0.68	0.65	-0.25
	Leucine	0.83	-0.07	0.36	0.98	0.13	0.11
	Tryptophan	0.86	0.18	0.40	0.88	0.01	0.43
	Serine	0.99	-0.06	-0.07	-0.64	0.21	0.15
	Threonine	0.99	-0.12	0.02	0.96	0.12	0.00
	Aspartic Acid	0.93	0.15	0.25	0.68	0.41	0.05
	Asparagine	0.38	-0.88	-0.25	0.89	0.40	0.08
	Valine	1.00	-0.01	-0.07	0.95	0.26	0.17
	Isoleucine	0.99	-0.09	0.05	0.96	0.24	0.08
Organic acids	Gamma-Hydroxybutyric Acid	-0.05	0.96	0.03	-0.31	-0.45	0.54
	Benzoic Acid	-0.20	0.91	-0.26	-0.33	0.67	0.53
	4-Hydroxy-Cinnamic Acid	0.97	0.19	0.06	0.93	0.17	-0.14
	Beta-D-Glucopyranuronic Acid	0.88	-0.12	0.37	-0.93	0.07	0.34
	Propanoic Acid	-0.34	0.52	-0.59	-0.56	0.27	-0.28
	Pyruvic Acid	0.06	-0.95	-0.17	-0.49	0.48	-0.18
	Oxalic Acid	-0.70	-0.42	0.48	0.76	0.38	0.49
	Erythronic Acid	0.97	-0.11	-0.04	-0.63	0.57	-0.37
	Eicosanoic Acid	0.57	0.62	0.49	0.08	-0.83	0.52
	Glyceric Acid	0.97	-0.01	-0.22	-0.36	-0.01	0.43
	Glutamic Acid	-0.05	-0.89	-0.39	0.77	0.40	-0.44
	Succinic Acid	0.55	0.30	-0.75	-0.73	0.59	-0.12
	Quinic Acid	0.92	-0.04	0.19	-0.08	0.39	0.81
	Phosphoric Acid	-0.66	-0.37	0.49	0.15	0.77	-0.31
	Citric Acid	0.33	-0.55	0.71	0.52	0.78	0.10
	Pipecolic Acid	0.91	0.41	-0.02	0.71	0.67	0.16
	Malic Acid	-0.50	0.03	-0.13	-0.76	0.62	0.12
	Gluconic Acid	0.88	0.40	-0.02	0.66	0.57	-0.17
	Octadecanoic Acid	-0.13	0.91	0.30	0.05	-0.83	0.54
	Hexadecanoic Acid	-0.03	0.92	0.34	0.18	-0.91	0.37
	Salicylic Acid	-0.25	-0.30	-0.16	0.01	0.59	0.63
	Threonic Acid	0.93	0.03	-0.35	0.14	-0.13	0.03
	Saccharic Acid	0.95	0.16	0.15	-0.51	0.53	-0.18
	3-Deoxy-Arabino-Hexaric Acid	0.97	-0.12	0.02	-0.46	0.81	0.29
	Octadecatrienoic Acid	-0.57	-0.28	-0.06	-0.34	0.39	0.86
	Fumaric Acid	0.94	0.33	0.01	-0.66	0.65	0.36
	Acetic Acid	0.79	0.23	-0.09	-0.45	0.84	-0.19
	Isocitric Acid	0.65	-0.11	0.69	0.55	0.73	-0.13
Sugars	Glucopyranose	0.71	-0.53	0.45	0.61	-0.22	-0.22
	Inositol-2-Phosphate	0.96	-0.25	0.10	0.12	0.96	0.13
	Fructofuranose	0.64	-0.54	0.51	0.40	-0.14	-0.35
	D-Xylofuranose	0.19	0.97	-0.04	-0.69	0.60	0.40
	Mannose	0.77	-0.51	0.27	0.59	0.44	0.54
	Mannitol	0.99	-0.12	-0.02	0.29	-0.53	-0.34
	Glycerol	0.89	-0.01	-0.45	-0.31	0.08	0.73

	Trehalose	0.82	-0.28	0.38	0.97	0.22	-0.08
	Inositol	0.99	-0.09	0.09	0.84	-0.09	0.50
	Lyxos	0.98	0.09	-0.10	0.62	0.63	0.13
	Maltose	0.95	0.29	0.00	-0.46	-0.18	0.47
	Raffinose	0.97	0.18	-0.15	0.43	-0.70	0.52
	Xylose	0.99	0.06	-0.10	-0.88	0.34	0.09
	Xylitol	0.65	-0.16	-0.62	-0.20	0.86	0.46
	Lactose	0.99	0.09	-0.10	0.12	-0.39	0.45
	Sorbitol	0.98	0.00	-0.11	0.22	-0.82	0.46
	Melezitose	0.86	-0.01	-0.49	0.60	0.27	0.26
	Pentitol	0.70	0.50	0.46	0.96	0.17	0.16
	Ethylene Glycol	-0.75	0.01	0.02	-0.69	0.69	0.21
	Sucrose	0.98	-0.14	-0.12	-0.09	-0.65	0.51
	Fructose	0.61	-0.64	0.35	0.52	-0.27	-0.56
	Glucose	0.69	-0.49	0.52	0.58	-0.28	-0.17
	Galactinol	0.90	0.07	0.43	-0.30	-0.53	0.77
	1-Ethylglucopyranoside	-0.71	-0.32	0.32	0.00	0.67	-0.05
	Salicylic Acid-Glucopyranoside	0.89	-0.05	0.17	-0.46	0.55	0.28
	1-Monohexadecanoylglycerol	0.50	0.84	0.13	0.32	-0.88	0.22
	1-Monooctadecanoylglycerol	0.48	0.86	0.14	0.37	-0.88	0.23
Others	2-Aminoethanol	0.95	0.08	-0.26	0.87	0.41	-0.08
	5-Hydroxy-Tryptamine	0.36	0.92	0.04	0.21	0.40	0.44
	Adenosine	0.93	-0.15	-0.02	-0.47	-0.14	0.45
	Allantoin	0.66	0.04	0.71	0.74	0.11	-0.31
	Campesterol	-0.54	-0.11	0.82	0.17	0.12	0.97
	Erythritol	0.83	-0.36	-0.18	-0.50	0.33	-0.42
	Ethanolanine	0.29	0.63	0.41	0.88	0.33	0.02
	Formylpiperidine	-0.48	0.30	-0.78	-0.23	0.42	0.80
	Galactosylglycerol	0.84	-0.16	-0.39	0.47	-0.59	0.58
	Threonic Acid-1,4-Lactone	0.80	-0.18	-0.56	0.26	0.56	-0.19
	Triethanolamine	0.44	-0.89	0.05	-0.30	-0.54	-0.39
	Uracil	0.72	0.43	-0.52	-0.36	-0.02	0.92
	Urea	0.89	-0.22	0.12	-0.04	-0.36	-0.59
	Vitamin E	-0.90	0.11	0.27	0.41	-0.77	0.07

Table S8. Significant fold changes (in concentration) of some metabolites in two rice genotypes (IR64 and PL177) in response to the salt and salt+ABA (SA) treatments at the seedling stage

			Salt/control		SA/control	
Tissue	Metabolites	Type*	PL177	IR64	PL177	IR64
Shoots	Alanine	I	3.6	3.2	2.7	4.0
Shoots	Glycine	I	4.2	5.4	3.6	5.4
Shoots	Tyrosine	I	3.9	4.7	4.3	4.1
Shoots	Serine	I	4.1	4.8	3.8	4.7
Shoots	Valine	I	4.7	5.1	4.5	4.4
Shoots	Isoleucine	I	4.4	5.1	4.8	4.9
Shoots	4-Amino-ButyricAcid	I	4.4	5.5	4.3	5.1
Shoots	ErythronicAcid	I	3.8	2.9	3.7	2.8
Shoots	GlycericAcid	I	4.1	4.8	3.3	4.0
Shoots	QuinicAcid	I	2.5	4.0	3.2	4.5
Shoots	Mannitol	I	2.8	4.3	2.8	4.4
Shoots	Maltose	I	4.4	5.3	5.0	3.2
Shoots	SalicylicAcid-Glucopyranoside	I	3.5	2.5	4.5	2.1
Shoots	Inositol,Myo-	I	4.4	4.2	4.9	4.5
**Shoots**	**Sorbitol**	**I**	**11.0**	**8.6**	**10.1**	**6.6**
Shoots	ThreonicAcid-1,4-Lactone	I	7.1	6.0	2.9	5.6
Shoots	Galactinol	I	3.3	3.4	6.2	3.3
Shoots	Allantoin	I	2.0	5.2	11.3	5.2
Shoots	Raffinose	I	17.2	19.6	15.2	12.2
**Shoots**	**Adenosine**	**I**	**18.5**	**44.9**	**18.7**	**40.8**
Shoots	Glucopyranose	II	5.1	4.2	9.0	19.5
Shoots	Mannose	II	6.1	5.3	7.7	16.0
Shoots	Leucine	II	4.1	5.3	7.1	4.6
**Shoots**	**Fructofuranose**	**II**	**12.3**	**6.2**	**27.4**	**58.6**
**Shoots**	**Fructose**	**II**	**14.8**	**28.1**	**22.3**	**515.1**
**Shoots**	**Glucose**	**II**	**6.0**	**4.5**	**12.7**	**22.4**
Shoots	Vitamin E	II	-24.9	-5.2	-2.1	-11.2
Shoots	Melezitose	II	16.4	14.2	7.7	9.5
Shoots	Xylose	II	9.6	18.9	9.0	13.4
Shoots	Lyxos	II	6.5	9.2	6.3	6.0
**Shoots**	**Glutamine**	**II**	**4.7**	**13.3**	**6.3**	**2.6**
Shoots	Galactosylglycerol	II	7.6	12.0	4.6	8.8
Shoots	ThreonicAcid	II	9.3	15.2	6.0	10.0
Shoots	Tryptophan	II	6.9	9.2	15.3	5.5
Shoots	Pentitol	II	3.4	11.5	22.0	4.7
**Shoots**	**Proline**	**II**	**66.0**	**203.8**	**25.3**	**768.9**
**Roots**	**Urea**	**II**	**-1.5**	**49.0**	**-1.4**	**30.3**
Roots	Phenylalanine	II	1.5	2.6	4.9	4.7
**Roots**	**Proline**	**II**	**2.8**	**8.4**	**3.3**	**15.9**

Roots	Glutamine	II	2.7	1.2	3.2	8.0
Roots	Arginine	II	2.6	1.7	4.2	5.3
**Roots**	**Asparagine**	**II**	**7.8**	**2.3**	**10.3**	**14.4**
Roots	Isoleucine	II	1.3	2.9	2.4	6.4
Roots	Trehalose	II	2.4	2.2	4.1	5.9
Roots	Pentitol	II	3.2	3.9	7.4	8.2
**Roots**	**Allantoin**	**II**	**111.9**	**-1.0**	**57.7**	**41.8**

*I = salt responsive + ABA insensitive, II = salt and ABA responsive (increase, decrease), respectively.

Table S9. List of primers of genes for the quantitative RT-PCR

Gene ID	Forward primer	Reverse primer
Os07g0638300	5' AGGCAGCACAGTCGTTCGT 3'	5' GGTTCCCAGGCTTGTAGGC 3'
Os01g0795000	5' TTGACTACGGAAGTGGAAACA 3'	5' GAACAGATGGCTCAACAACC 3'
Os02g0569900	5'CCGAGAAGTTCAAGTCTGCCAATG3'	5' CGTGCCACTGGGTCCCGTAT 3'
Os05g0381400	5' ACGAGATGGCCGGAGTAGG 3'	5' GGATGGCGAAGACGAGGAAG 3'
Os12g0259800	5' TCATTGCCATTGCTTTGGTTTCT 3'	5' CTGGCAAAGTTGGCTGATGGA 3'
Os05g25350.1	5' ACCTATGATACACCCAAGCC 3'	5' TTGGGAAGGAATAGGTGTAA 3'
Os09g0248900	5' AGGTAGAACCGCAGGAGA 3'	5' GATACACGGTAACGAGGG 3'
Os05g0477600	5' GGCTATGCTGAGGCTGCTA 3'	5' CCCAAACAATACAGGAACAGA 3'
Os12g0555000	5' ACCCTGCTGTGGATGATGG 3'	5' AGATTGGATTTGTCGTGGC 3'
Os07g0561800	5' GCCTCCAACTCGCATCTT 3'	5' AGTTCGGAGTTACTACTACTGGTT 3'

Table S10. Common DEGs detected in both shoots and roots of both genotypes under salt stress

Type	Probe Set ID	Gene name	Shoots		Roots		Function Annotation
			PL177	IR64	PL177	IR64	
I	Os.51718.1.S1	Os11g0454200	117.9	101.3	9.4	8.9	Dehydrin RAB 16B.
	Os.11260.1.S1	Os05g0381400	88.4	61.4	18.8	14.3	ABA induced plasma membrane protein PM 19.
	OsAffx.18737.1.S1	Os11g0181200	51.7	30.3	5.7	6.2	Hypothetical protein.
	Os.12415.1.S1	Os09g0109600	51.5	51.5	2.5	2.2	Conserved hypothetical protein.
	Os.9988.1.S1	Os11g32890	38.6	43.2	4.8	4.6	expressed protein
	Os.51165.1.S1	Os11g0303600	36.0	27.6	3.0	2.5	O-methyltransferase, family 2 domain containing protein.
	Os.49245.1.S1	Os02g0649300	29.1	37.6	13.0	13.8	Short highly repeated, interspersed DNA (Fragment).
	Os.12633.1.S1_s	Os11g0454300	27.8	48.1	18.4	11.0	Dehydrin family protein.
	Os.11491.1.S1	Os03g0305600	24.0	26.1	2.4	2.0	Mitochondrial import inner membrane translocase
	Os.56875.1.S1	Os08g0442900	18.7	21.2	8.7	9.6	Conserved hypothetical protein.
	Os.46582.1.S1	Os10g0505900	24.9	19.0	2.5	2.7	Conserved hypothetical protein.
	Os.9103.1.S1	Os09g0272000	12.0	10.5	2.2	2.1	Heavy metal transport/detoxification protein domain containing protein.
	Os.51752.2.S1_x	Os05g0170200	10.1	11.3	2.9	3.2	Conserved hypothetical protein.
	Os.27138.1.S1	Os10g0360100	9.0	9.7	2.7	4.1	Sugar transporter protein.
II	Os.3280.1.S1	Os10g0548100	29.2	21.9	37.1	38.9	Conserved hypothetical protein.
	OsAffx.26317.1.S2	Os04g0415800	27.4	28.9	29.7	26.7	Plant lipid transfer/seed storage/trypsin-alpha amylase inhibitor domain containing protein.
*	Os.5325.1.S1	Os08g0327700	24.9	12.3	12.4	11.6	Late embryogenesis abundant (LEA) group 1 family protein.
	Os.53210.1.S1	Os11g0454000	14.4	9.1	8.2	7.3	Dehydrin family protein.
	Os.39552.1.A1_s	Os06g0698300	14.1	14.1	3.3	3.1	Protein phosphatase 2C family protein.
	Os.51775.1.S1	Os12g0147200	12.1	11.3	12.0	10.8	Hypothetical protein.
	Os.55564.1.S1	Os02g0158900	11.4	8.4	9.3	8.5	SNF4.
*	Os.12551.1.S1_s	Os05g0542500	10.0	20.1	10.4	7.8	Group 3 LEA (Type I) protein.
	Os.55197.1.S1	Os06g0341300	9.6	8.1	6.3	5.3	Seed maturation protein domain containing protein.
	Os.39000.1.S1	unknown	9.5	8.8	5.1	5.3	Putative uncharacterized protein
	Os.2544.1.S1_s	Os10g0492600	8.5	6.2	2.8	2.7	Tonoplast membrane integral protein ZmTIP3-1.
	Os.33968.1.S1	Os01g0385400	7.9	6.5	2.5	2.1	C4-dicarboxylate transporter/malic acid transport protein family protein.
	Os.8559.1.S1	Os06g0651200	6.9	7.4	5.2	4.7	Hypothetical protein.

	Os.54410.1.S1	Os06g0246500	6.8	5.4	5.6	4.5	Pyruvate dehydrogenase E1 alpha subunit
	Os.51394.1.S1	Os05g0171200	6.8	5.5	3.8	3.5	Embryo-specific 3 family protein.
	Os.4867.1.S1	Os08g0518800	6.6	5.5	3.1	3.6	Chitinase (EC 3.2.1.14).
	Os.28617.1.S1	Os02g0824500	6.3	6.2	4.3	2.9	Remorin.
III	Os.9820.1.S1	Os11g0453900	18.3	14.2	26.0	23.8	Dehydrin RAB 16D.
	Os.6274.1.S1	Os05g0550600	12.1	10.3	20.6	23.2	Nonspecific lipid transfer protein.
	Os.54579.1.S1_s	Os07g0418700	5.7	4.2	10.5	10.7	Conserved hypothetical protein.
IV	OsAffx.6397.1.S1	Os09g0425200	2.0	3.7	9.8	4.6	HMW kininogen family protein.
	Os.9022.1.S1	Os09g0325700	5.3	4.5	4.2	3.3	Protein phosphatase 2C
	Os.50053.1.A1	Os07g0154100	4.8	3.5	3.4	6.4	Viviparous-14.
	Os.54469.1.S1	Os01g0844300	4.7	5.1	10.1	8.7	Peptidylprolyl isomerase.
	Os.29815.1.S1	Os01g0962100	4.6	4.2	2.7	2.6	Conserved hypothetical protein.
	Os.18257.1.S1	Os07g0209100	4.3	4.4	2.8	2.6	Raffinose synthase family protein.
	Os.38581.1.S1_s	Os05g0460000	4.2	3.7	5.6	3.9	Heat shock protein 70.
	Os.53614.1.S1	Os02g0755900	4.1	3.9	4.8	4.1	UDP-glucuronosyl/UDP-glucosyltransferase family protein.
	Os.12725.1.S1	Os06g0160700	3.9	3.4	3.0	2.6	Starch synthase I, chloroplast precursor
	Os.28435.1.S1_a	Os10g0558700	3.8	5.0	3.6	3.4	2OG-Fe(II) oxygenase domain containing protein.
	Os.12693.3.S1	Os07g0694700	3.6	3.6	2.4	2.1	Ascorbate peroxidase (EC 1.11.1.11).
	Os.39973.1.S1_s	Os03g0820500	3.6	4.3	7.8	5.7	Actin-binding, cofilin/tropomyosin type domain containing protein.
	Os.8971.1.S1	Os05g0519700	3.5	3.1	3.1	2.4	101 kDa heat shock protein.
	Os.38021.1.S1	Os07g0633200	3.5	2.8	2.0	2.3	Serine/arginine-rich protein
	Os.459.1.S1	Os03g0663500	3.5	4.2	6.9	6.5	Thaumatin, pathogenesis-related family protein.
	Os.46021.1.S1	Os01g0654400	3.3	3.6	3.6	2.6	Conserved hypothetical protein.
	Os.53085.1.S1	Os05g0574600	3.2	3.7	2.4	2.1	Fatty acid elongase 1-like protein.
	Os.55479.1.S1	Os05g0494600	3.1	2.6	5.3	3.2	Conserved hypothetical protein.
	Os.38157.1.S1	Os09g0572400	3.1	3.9	2.1	2.1	ATP-binding cassette, sub-family F
	Os.28952.1.S1	Os01g0183600	3.0	2.5	2.6	2.2	Cytochrome P450 family protein.
	Os.13596.1.S1	Os03g0200200	3.0	3.7	3.8	3.0	Hypothetical protein.
	Os.22928.1.S1_x	Os02g0580900	2.9	2.9	2.2	2.4	TGF-beta receptor, type I/II extracellular region family protein.
	Os.53117.1.S1_x	Os12g0478200	2.9	3.1	3.0	2.7	GRAM domain containing protein.
	Os.1465.1.S1	Os01g0190000	2.9	2.2	2.8	2.5	Taurine catabolism dioxygenase TauD/TfdA family
	Os.4530.1.S1	LOC_Os09g31482	2.7	2.8	2.6	2.4	splicing factor U2af 38 kDa subunit, putative, expressed

	Os.55494.1.S1	Os12g0153200	2.6	3.1	3.1	3.2	Hypothetical protein.
	Os.53414.1.S1	Os03g0287400	2.6	5.1	3.7	2.7	LOB domain protein 11.
	Os.54615.1.S1	Os06g0289200	2.5	2.4	5.7	5.6	UDP-glucuronosyl/UDP-glucosyltransferase family protein.
	Os.11510.1.S1	Os09g0491100	2.5	2.6	2.6	2.9	Beta-primeverosidase (EC 3.2.1.149).
	Os.52136.1.S1	Os03g0808900	2.3	2.6	2.5	2.7	Seed imbibition protein.
	Os.27430.1.S1	unknown	2.3	2.2	4.8	4.5	Unknown
	Os.17419.1.S1	Os10g0177200	2.3	2.0	3.8	3.9	Calcium-binding EF-hand domain containing protein.
	Os.11040.1.S1_x	Os10g0450800	2.2	2.0	2.4	2.4	Glycine-rich cell wall structural protein 2 precursor.
	Os.5087.1.S1	Os11g0132700	2.2	2.7	2.6	2.3	Cytochrome cd1-nitrite reductase-like
	Os.35154.1.S1_x	Os04g0513400	2.1	2.0	4.1	3.6	Beta-glucosidase.
	Os.2416.1.S1_a	Os01g0940700	2.0	2.9	5.7	5.0	Beta-1,3-glucanase (Fragment).
V	Os.27483.1.S1	Os08g0231400	3.2	2.1	0.4	0.4	Germin family protein.
	Os.11465.1.S1	Os11g0539200	2.0	2.5	0.4	0.3	Glycoside hydrolase, family 16 domain containing protein.
VI	Os.50995.1.S1	Os01g0696800	0.32	0.23	7.2	8.5	Peptidase A1, pepsin family protein.
	Os.2373.1.S1	Os10g0555700	0.46	0.37	2.1	2.3	Major pollen allergen Lol pI family protein.
	OsAffx.15178.1.S1_s	Os05g0579600	0.25	0.25	6.0	4.1	Myb, DNA-binding domain containing protein.
	Os.5031.1.S1	Os12g0555000	0.23	0.20	3.1	2.2	Bet v I allergen family protein.
	Os.3391.1.S1	Os09g0401000	0.23	0.20	4.2	2.7	Myb-related protein Pp2.
	Os.39087.1.S1	Os01g0248300	0.42	0.43	2.4	2.1	Conserved hypothetical protein.
	Os.2423.1.S1	Os12g0555200	0.21	0.26	2.5	2.8	Probenazole-inducible protein PBZ1.
	Os.19375.1.S1	Os08g0447900	0.15	0.13	2.2	2.2	Adenylyl cyclase associated protein/4-coumarate--CoA ligase 1
	Os.15139.1.S1	Os07g0417200	0.15	0.17	4.6	4.4	Delta-12 oleate desaturase.
	Os.5242.1.S1	Os01g0795000	0.11	0.10	6.3	5.0	Subtilisin-like secreted protease.
	Os.23207.1.S1	Os08g0137800	0.09	0.07	2.1	2.3	Plastocyanin-like domain containing protein.
	Os.23606.1.S1	Os01g0389700	0.07	0.06	2.4	3.0	Protein of unknown function DUF679 family protein.
	OsAffx.14446.1.S1_s	Os04g0664600	0.05	0.05	2.2	2.1	Agmatine coumaroyltransferase.
VII	Os.35196.1.S1	Os05g0589400	0.45	0.34	0.38	0.28	I-box binding factor (Fragment).
	Os.6114.1.S1	Os05g0187100	0.45	0.36	0.45	0.46	Hexokinase.
	Os.20169.1.S1	Os06g0538400	0.42	0.32	0.38	0.34	Hypothetical protein.
	OsAffx.4084.1.S1	Os04g0523700	0.40	0.29	0.20	0.18	UDP-glucuronosyl/UDP-glucosyltransferase family protein.
	Os.7240.2.S1_a	Os01g0749400	0.38	0.34	0.44	0.50	Glycosyl transferase, family 20 domain containing protein.
	Os.42069.1.S1_x	Os01g0760000	0.37	0.30	0.15	0.35	Dynein 8 kDa light chain, flagellar outer arm.

Os.26472.1.S1	Os04g0482300	0.33	0.35	0.20	0.26	TAZ finger domain containing protein.
Os.7344.1.S1	Os02g0756600	0.33	0.29	0.42	0.42	Phosphate-induced protein 1 conserved region family protein.
Os.10486.1.S1	Os01g0642200	0.32	0.26	0.08	0.08	Conserved hypothetical protein.
Os.7228.1.S1	Os02g0756200	0.32	0.25	0.33	0.43	Phi-1 protein.
Os.42024.1.S1	Os01g0770700	0.29	0.29	0.41	0.38	Copper transporter 1.
Os.8802.1.S1	Os02g0327500	0.26	0.24	0.19	0.27	Arabidopsis protein of unknown function DUF266 family protein.
Os.26792.1.S1	Os11g0569600	0.26	0.22	0.26	0.27	Protein kinase domain containing protein.
Os.26932.1.S1	Os07g0559700	0.24	0.21	0.27	0.30	Monosaccharide transporter 3.
Os.8481.1.S3_a	Os03g0237500	0.24	0.21	0.40	0.34	MAP65/ASE1 family protein.
Os.48082.1.S1	Os09g0417800	0.21	0.13	0.25	0.32	DNA-binding WRKY domain containing protein.
Os.6683.1.S1	Os01g0793800	0.20	0.15	0.08	0.11	Conserved hypothetical protein.
Os.50198.1.S1	Os12g0592900	0.12	0.12	0.37	0.48	Hypothetical protein.
Os.27297.1.A1_s	Os08g0202300	0.11	0.15	0.30	0.28	leucine-rich repeat family protein / protein kinase family protein
Os.1503.1.S1	Os06g0662200	0.09	0.09	0.20	0.18	Basic-leucine zipper (bZIP) transcription factor domain containing protein.
Os.2881.1.S1	Os10g0550900	0.09	0.08	0.16	0.22	Proline dehydrogenase domain containing protein.

* Significant difference was detected between the two genotypes.

Table S11. GO enrichment analysis of the PL177-specific DEGs under salt stress

			GO Information	Shoots-Up		Shoots-Down		Roots-Up		Roots-Down	
**No**	**GO Term**	**Onto**	**Description**	**FDR**	**Num**	**FDR**	**Num**	**FDR**	**Num**	**FDR**	**Num**
1	GO:0005976	P	polysaccharide metabolic process	0.029	8	---	---	---	---	---	---
2	GO:0016798	F	hydrolase activity, acting on glycosyl bonds	0.0024	9	---	---	---	---	---	---
3	GO:0004553	F	hydrolase activity, hydrolyzing O-glycosyl compounds	0.0024	8	---	---	---	---	---	---
**4**	**GO:0031982**	**C**	**vesicle**	**1.2E-07**	**79**	---	---	---	---	---	---
**5**	**GO:0016023**	**C**	**cytoplasmic membrane-bounded vesicle**	**1.2E-07**	**79**	---	---	---	---	---	---
**6**	**GO:0031988**	**C**	**membrane-bounded vesicle**	**1.2E-07**	**79**	---	---	---	---	---	---
**7**	**GO:0031410**	**C**	**cytoplasmic vesicle**	**1.2E-07**	**79**	---	---	---	---	---	---
8	GO:0005576	C	extracellular region	0.0042	6	---	---	---	---	---	---
9	GO:0034641	P	cellular nitrogen compound metabolic process	---	---	0.016	13	---	---	---	---
**10**	**GO:0016301**	**F**	**kinase activity**	**---**	**---**	**0.0029**	**27**	---	---	---	---
**11**	**GO:0016773**	**F**	**phosphotransferase activity, alcohol group as acceptor**	**---**	**---**	**0.0057**	**23**	---	---	---	---
12	GO:0005506	F	iron ion binding	---	---	0.0077	12	---	---	---	---
13	GO:0046906	F	tetrapyrrole binding	---	---	0.012	8	---	---	---	---
14	GO:0004674	F	protein serine/threonine kinase activity	---	---	0.012	18	---	---	---	---
15	GO:0004672	F	protein kinase activity	---	---	0.021	19	---	---	---	---
**16**	**GO:0003824**	**F**	**catalytic activity**	**---**	**---**	**0.022**	**84**	**---**	**---**	**---**	**---**
**17**	**GO:0016772**	**F**	**transferase activity, transferring phosphorus-containing groups**	**---**	**---**	**0.022**	**30**	---	---	---	---
18	GO:0020037	F	heme binding	---	---	0.023	7	---	---	---	---
**19**	**GO:0016740**	**F**	**transferase activity**	**---**	**---**	**0.039**	**41**	---	---	---	---
20	GO:0030554	F	adenyl nucleotide binding	---	---	0.046	18	---	---	---	---
21	GO:0001883	F	purine nucleoside binding	---	---	0.046	18	---	---	---	---
22	GO:0001882	F	nucleoside binding	---	---	0.046	18	---	---	---	---
23	GO:0034357	C	photosynthetic membrane	---	---	6.3E-09	15	---	---	---	---
24	GO:0042651	C	thylakoid membrane	---	---	6.3E-09	14	---	---	---	---

25	GO:0009579	C	thylakoid	---	---	8.5E-09	16	---	---	---	---
26	GO:0055035	C	plastid thylakoid membrane	---	---	8.5E-09	13	---	---	---	---
27	GO:0009535	C	chloroplast thylakoid membrane	---	---	8.5E-09	13	---	---	---	---
28	GO:0044436	C	thylakoid part	---	---	3.0E-08	14	---	---	---	---
29	GO:0009534	C	chloroplast thylakoid	---	---	7.1E-08	13	---	---	---	---
30	GO:0031976	C	plastid thylakoid	---	---	8.9E-08	13	---	---	---	---
31	GO:0031984	C	organelle subcompartment	---	---	1.0E-07	13	---	---	---	---
32	GO:0031090	C	organelle membrane	---	---	2.7E-07	19	---	---	---	---
33	GO:0044434	C	chloroplast part	---	---	1.3E-06	14	---	---	---	---
34	GO:0044435	C	plastid part	---	---	2.9E-06	14	---	---	---	---
**35**	**GO:0009507**	**C**	**chloroplast**	**---**	**---**	**6.5E-06**	**22**	---	---	---	---
**36**	**GO:0044422**	**C**	**organelle part**	**---**	**---**	**2.8E-03**	**26**	---	---	---	---
**37**	**GO:0044446**	**C**	**intracellular organelle part**	**---**	**---**	**2.8E-03**	**26**	---	---	---	---
**38**	**GO:0016020**	**C**	**membrane**	**---**	**---**	**8.7E-03**	**55**	---	---	---	---
39	GO:0015979	P	photosynthesis	---	---	---	---	8.5E-04	6	---	---
40	GO:0032787	P	monocarboxylic acid metabolic process	---	---	---	---	8.5E-04	10	---	---
41	GO:0016491	F	oxidoreductase activity	---	---	---	---	1.1E-03	15	---	---
42	GO:0004497	F	monooxygenase activity	---	---	---	---	2.4E-02	5	---	---

Note: GO enrichment analysis was performed using AgriGO (http://bioinfo.cau.edu.cn/agriGO/), GO terms were significantly enriched in the lists of those specifically up- and down-regulated genes in the 177-103 shoot and root under salt stress treatment (Fold change≧2, p≦0.05).

Table S12. List of genes that were exclusively expressed in PL177 that were all mapped on the introgressed chromosome regions of PL177

Probe Set ID	Name	PL177-Shoot	PL177-Roots	Chr.	Description
Os.7705.1.S1	LOC_Os04g54300		2.02	4	wound induced protein, putative, expressed
**Os.7825.1.S1**	**Os04g0105100**		**2.04**	**4**	**Zn-finger, RING domain containing protein.**
Os.18124.1.S1_x	Os04g0126900	2.72		4	Conserved hypothetical protein.
OsAffx.3726.1.S1	Os04g0137100	2.13		4	Pectate lyase (Fragment).
OsAffx.25891.1.S1	Os04g0150300	3.79		4	Conserved hypothetical protein.
OsAffx.15214.1.S1_x	Os06g0103800	2.12		6	Protein of unknown function DUF125
Os.5600.1.S1_s	Os06g0127500	2.03		6	MEG5.
Os.12784.1.S1	Os06g0130800	2.11		6	Histone H5 family protein.
Os.20157.1.A1	Os06g0132500	2.70	2.25	6	Alpha/beta hydrolase family protein.
Os.4773.1.S1	Os06g0133500		2.17	6	Conserved hypothetical protein.
Os.46584.1.S1	Os06g0143100		2.62	6	Hypothetical protein.
Os.46583.2.S1	Os06g0143900	2.04		6	Coatomer protein complex, beta prime
Os.50550.1.S1	Os06g0148600	2.02		6	Cyclin-like F-box domain containing protein.
Os.4223.1.S1_s	Os06g0168700	4.50	3.47	6	Prolin rich protein.
Os.54299.1.S1	Os06g0211200	2.32	2.44	6	ABA-responsive element binding protein (AREB1).
Os.15957.1.S1	Os06g0221200	2.01		6	Annexin p33.
**Os.9950.1.S1**	**Os06g0253100**	**2.33**		**6**	**Heat shock protein Hsp20**
Os.51106.1.S1	Os06g0256900	2.03		6	Endo-beta-1,4-glucanase precursor
Os.47737.1.S1	Os06g0257600		2.31	6	Lipolytic enzyme, G-D-S-L family protein.
Os.47521.1.A1	Os06g0260500	2.87		6	Naringenin-chalcone synthase family protein.
Os.50548.2.S1_x	Os09g0345000		2.01	9	Conserved hypothetical protein.
Os.9782.1.S1	Os09g0363900	3.27		9	Mandelonitrile lyase-like protein.
Os.17577.1.S2	Os09g0381400		2.94	9	Peptidase C1A, papain family protein.
Os.52863.1.S1	Os09g0388400	3.90	0.46	9	Cof protein family protein.
Os.9685.1.S1_a	Os09g0419200		2.17	9	Cinnamoyl-CoA reductase.
OsAffx.20172.1.S1_x	Os10g0530700	2.12		10	Glutathione S-transferase GST 38
Os.46858.1.S1	Os10g0532200		2.07	10	Alpha/beta hydrolase family protein.
Os.46548.2.S1	Os10g0546600		2.07	10	Chloroplast carotenoid epsilon-ring hydroxylase.
OsAffx.18586.1.S1	Os10g0547900	2.01		10	Short-chain dehydrogenase Tic32.
Os.46632.1.S1	LOC_Os10g39800		2.12	10	hypothetical protein
Os.26482.1.S1	Os10g0551700	2.75		10	Plant lipid transfer
Os.46052.1.S1	Os10g0551900		2.54	10	Plant lipid transfer
OsAffx.30731.1.S1	LOC_Os10g40120		2.14	10	Hypothetical protein
Os.2404.1.S1	Os10g0555600	2.10		10	Beta-expansin precursor.
Os.46623.1.S1	Os10g0557900	2.92		10	92 kDa type IV collagenase precursor
Os.23290.1.S1	Os10g0558900		2.20	10	2OG-Fe(II) oxygenase domain containing protein.
Os.52326.1.S1	Os10g0559200	2.99		10	Conserved hypothetical protein.
Os.46540.1.S1	Os10g0560000	3.32		10	Plant protein of unknown function DUF828
Os.18989.1.S1	LOC_Os10g05660.4		2.26	10	Putative thaumatin-like protein
Os.25164.1.A1	LOC_Os11g22250	2.04		11	expressed protein
**OsAffx.30855.1.S1**	**LOC_Os11g07140**	**2.02**		**11**	**receptor kinase-like protein, putative, expressed**
OsAffx.31356.1.S1	LOC_Os11g37880		2.11	11	stripe rust resistance protein Yr10
Os.152.1.S1	Os11g0104400		2.09	11	Fatty acid desaturase subdomain containing protein.
